# Excitation‐Dependent Quadruple‐Level Emission from an Isolated Molecule for Dynamic Information Encryption

**DOI:** 10.1002/advs.202508987

**Published:** 2025-07-11

**Authors:** Yibo Shi, Lin Liu, Wei‐Hai Fang, Qian Wang, Xiao Liu, Kai Feng, Wei Sun, Dongpeng Yan, Xuebo Chen

**Affiliations:** ^1^ Key Laboratory of Theoretical and Computational Photochemistry Ministry of Education College of Chemistry Beijing Normal University Beijing 100875 China; ^2^ College of Chemistry and Molecular Sciences Henan University Zhengzhou 450046 China

**Keywords:** anti‐kasha emission, excitation‐dependent emission, multi‐dimensional information encryption, NEVPT2, room‐temperature phosphorescence

## Abstract

Stimuli‐responsive single‐molecule multi‐emission materials have long attracted considerable attention due to their great potential in non‐phase‐separated smart luminescence. Here, a new strategy is demonstrated for manipulating electron transfer based on donor‐acceptor decoupling to regulate energy levels, aiming to achieve excitation‐dependent (Ex‐De) single‐molecule emission with switchable multiple fluorescence and phosphorescence. The synthesized 10‐phenyl‐10H,13'H‐spiro[acridine 9,6'‐pentacen]‐13'‐one (ACRSP) exhibits anti‐Kasha quadruple‐level emission and opposite Ex‐De afterglow in different environments. The high‐energy emission bands of multi‐fluorescence in solution respond to excitation, whereas in poly(methyl methacrylate) (PMMA), phosphorescence‐fluorescence multi‐emission causes Ex‐De to appear in the low‐energy emission band. Experimental and computational results indicate that exciton spin ratios and emissive state compositions vary with excitation modes, leading to dual Ex‐De behavior from three fluorescence and one phosphorescence emissions. Donor‐acceptor decoupling separates locally excited (LE) and charge transfer (CT) states, while triplet level inversion enables Ex‐De behavior and room‐temperature phosphorescence (RTP) coexistence (τ = 770.54 ms). By tuning the excitation mode of ACRSP, we achieve Ex‐De long afterglow emission from an isolated molecule, enabling time‐resolved and excitation‐responsive multi‐dimensional information encryption. This work offers design guidelines for purely organic Ex‐De systems and paves the way for next‐generation single‐molecule responsive luminophores.

## Graphical abstract

By manipulating the quadruple decoupled radiative transitions of donor and acceptor units, dual‐phase excitation‐dependent afterglow emission is achieved for the first time in a single isolated molecule. The decoupled donor‐acceptor units precisely manipulate the electron transfer pathways in ACRSP, endowing it with intrinsic single‐molecule Ex‐De properties, resulting in anti‐Kasha quadruple emission levels. The precise control over exciton spin ratios and excited‐state components enables time‐resolved and wavelength‐responsive multidimensional information encryption.

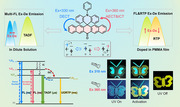

## 1 Introduction

Stimuli‐responsive multi‐emissive materials have attracted significant attention due to their unique photophysical properties,^[^ ^1^ ^]^ enabling responses to external environmental factors such as temperature,^[^ ^2^ ^]^ mechanical force,^[^ ^3^ ^]^ light,^[^ ^4^ ^]^ and pH.^[^ ^5^ ^]^ These materials have found wide applications in information encryption and signal transmission.^[^ ^6^ ^]^ Among them, excitation‐dependent (Ex‐De) emission shows great potential owing to its non‐invasive, non‐contact, remotely controllable, and highly selective characteristics.^[^ ^7^ ^]^ Incorporating Ex‐De into ultralong organic room‐temperature phosphorescence (RTP) systems enables multi‐level anti‐counterfeiting through dual modulation in both color and time domains, offering promising application prospects.^[^ ^1^, ^7^ ^]^

Most reported Ex‐De RTP systems rely on physical mixtures of multiple components,^[^ ^8^ ^]^ aggregation‐induced effects,^[^ ^9^ ^]^ or intrinsic polymerization,^[^ ^10^ ^]^ which may suffer from issues such as phase separation, color aging, and limited tunability.^[^ ^11^ ^]^ In contrast, single‐molecule Ex‐De systems offer improved structural stability but also face greater constraints. These limitations arise from three main challenges. First, Ex‐De emission typically requires the involvement of multiple excited states, including high‐energy singlet and triplet states that violate Kasha's rule, which are difficult to control through conventional molecular design.^[^ ^7^, ^12^ ^]^ Second, RTP in purely organic systems relies on highly rigid molecular frameworks to suppress non‐radiative decay, extending the T_1_ state lifetime to the millisecond or even second scale.^[^ ^13^ ^]^ However, such stabilization often compromises the flexible energy‐level tuning required for Ex‐De emission. Third, the inherently low intersystem crossing (ISC) efficiency in organic molecules, compared to metal complexes, results in weak phosphorescence signals that are typically difficult to detect via steady‐state spectroscopy or direct visual observation.^[^ ^14^ ^]^ These intrinsic contradictions present a formidable challenge for integrating Ex‐De behavior and RTP within an isolated molecular system.

To address this problem, decoupled donor‐acceptor (D‐A) molecular frameworks have recently emerged as promising candidates. In particular, spiro‐linked D‐A structures exhibit orthogonal geometries that spatially separate frontier molecular orbitals, allowing precise control over intramolecular charge transfer (CT) and locally excited (LE) states.^[^ ^15^ ^]^ These structures have been widely used in thermally activated delayed fluorescence (TADF) materials and have achieved excellent electroluminescent performance.^[^ ^15^, ^16^ ^]^ Inspired by this design concept, we aim to modularly tune the triplet‐state properties of the spiro structure to realize ultralong organic RTP alongside Ex‐De multi‐emission behavior.

**Figure 1:**
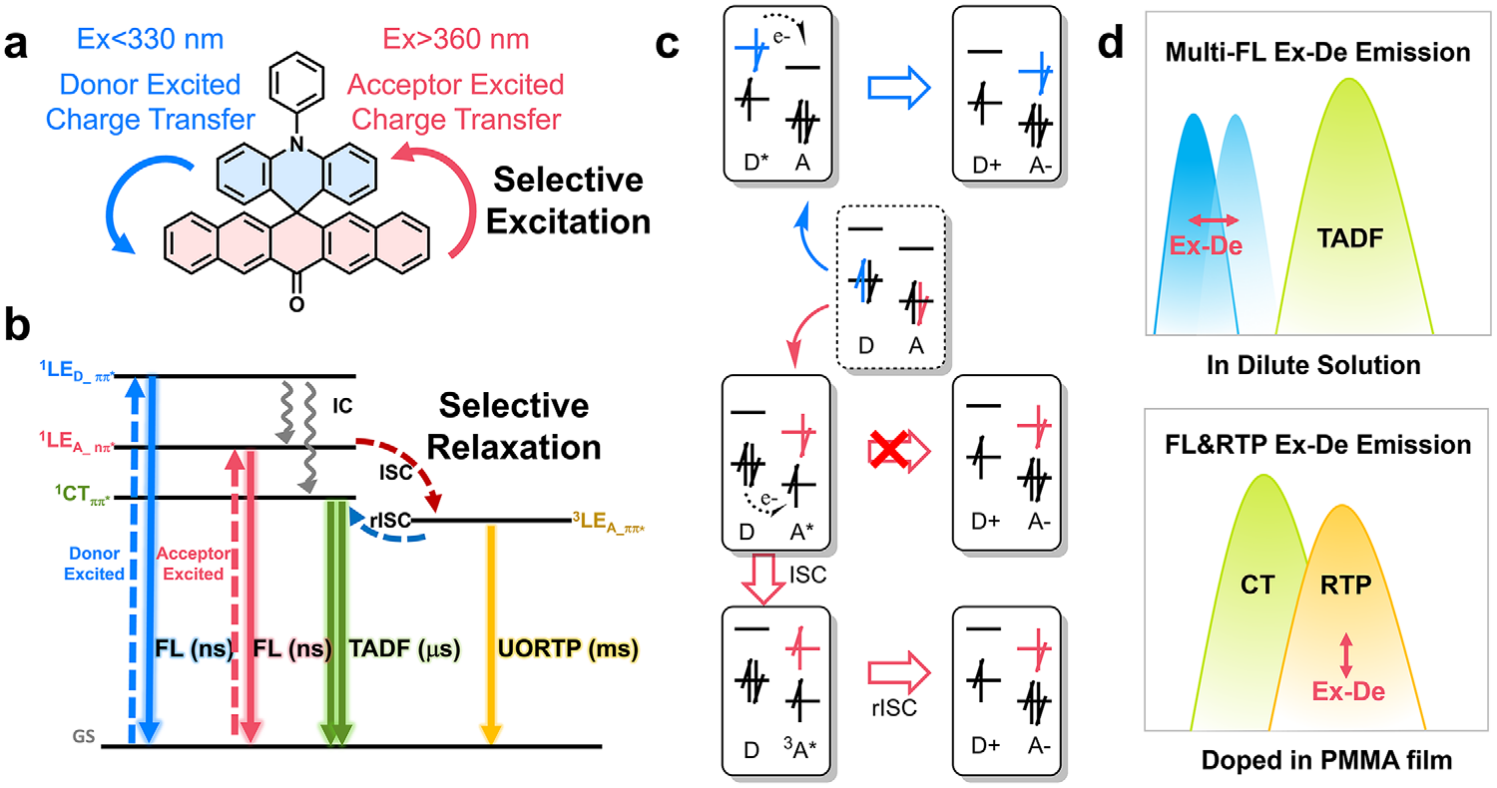
Schematic illustration of Ex‐De quadruple‐level emission. a) Molecular structure of ACRSP and its donor‐acceptor excitation modes. b) Jablonski diagram illustrating quadruple‐level emission of ACRSP. c) Multistep electron transfer and fluorescence‐phosphorescence generation pathways under excitation‐mode separation, originating from donor excited charge transfer (DECT) and acceptor excited charge transfer (AECT) processes. d) Schematic illustration of dual‐phase Ex‐De afterglow behavior.

In this work, we present a strategy of excitation mode separation coupled with energy level inversion to regulate the non‐radiative rate of high‐energy excited states while simultaneously tuning the emissive state properties within a donor‐acceptor decoupled system. The Ex‐De quadruple‐level emission was achieved in ACRSP (**Figure** **1**a), which includes three fluorescence emissions and one RTP (Figure 1b), marking the highest record for single‐molecule multi‐level emission at room temperature. Theoretical and experimental studies reveal that Ex‐De emission originates from the decoupled excitations of the donor and acceptor, leading to distinct singlet and triplet electron transfer processes (Figure 1c). The competition between electron transfer and exciton recombination enables ultra‐multi‐level Ex‐De emission. The Ex‐De emission of ACRSP varies across different phases (Figure 1d). In dilute solvents, ACRSP exhibits Ex‐De LE fluorescence and TADF due to rapid reverse intersystem crossing (rISC). In the rigid matrix, the destruction of the n‐π* state slows down nonradiative decay, resulting in the simultaneous expression of LE phosphorescence and CT fluorescence, with an RTP lifetime of up to 770.54 ms. The variation in excitation wavelength affects the triplet exciton population, thereby influencing the fluorescence‐phosphorescence ratio. This unique dual‐mode Ex‐De afterglow enables the precise modulation of three LE and one CT emissions by adjusting the excitation wavelength and environmental polarity. Leveraging the unique stimulus‐responsive multi‐level emission of the ACRSP system, we successfully demonstrate its potential applications in multi‐level information encryption and dynamic printing. This work aims to achieve tunable luminescence with extended emission lifetimes and enriched color variations, enabling the development of stimulus‐responsive single‐molecule multi‐level anti‐counterfeiting applications.

## 2 Results and Discussion

### 2.1 Synthesis and Structural Characterizations

In this study, we designed 10‐phenyl‐10H,13'H‐spiro[acridine‐9,6'‐pentacen]‐13'‐one (ACRSP) using pentacene ketone as the acceptor, connected via a spiro‐σ‐bridge to a phenyl acridine donor unit. As shown in Figure S11 (Supporting Information), ACRSP was synthesized on a gram scale starting from 6,13‐pentacenedione and 2‐bromotriphenylamine via lithium‐halogen exchange and dehydrative cyclization, with an overall yield of 28%. For comparison, 10‐phenyl‐10H,10'H‐spiro[acridine‐9,9'‐anthracen]‐10'‐one (ACRSA) was synthesized through a similar pathway. The molecular structures and purities were confirmed by ^1^H NMR, ^13^C NMR, high‐resolution mass spectrometry (HRMS), high‐performance liquid chromatography (HPLC), and single‐crystal X‐ray diffraction. Detailed synthetic and characterization procedures are provided in the Supporting Information (Figures S11 and S21, Supporting Information).

**Figure 2:**
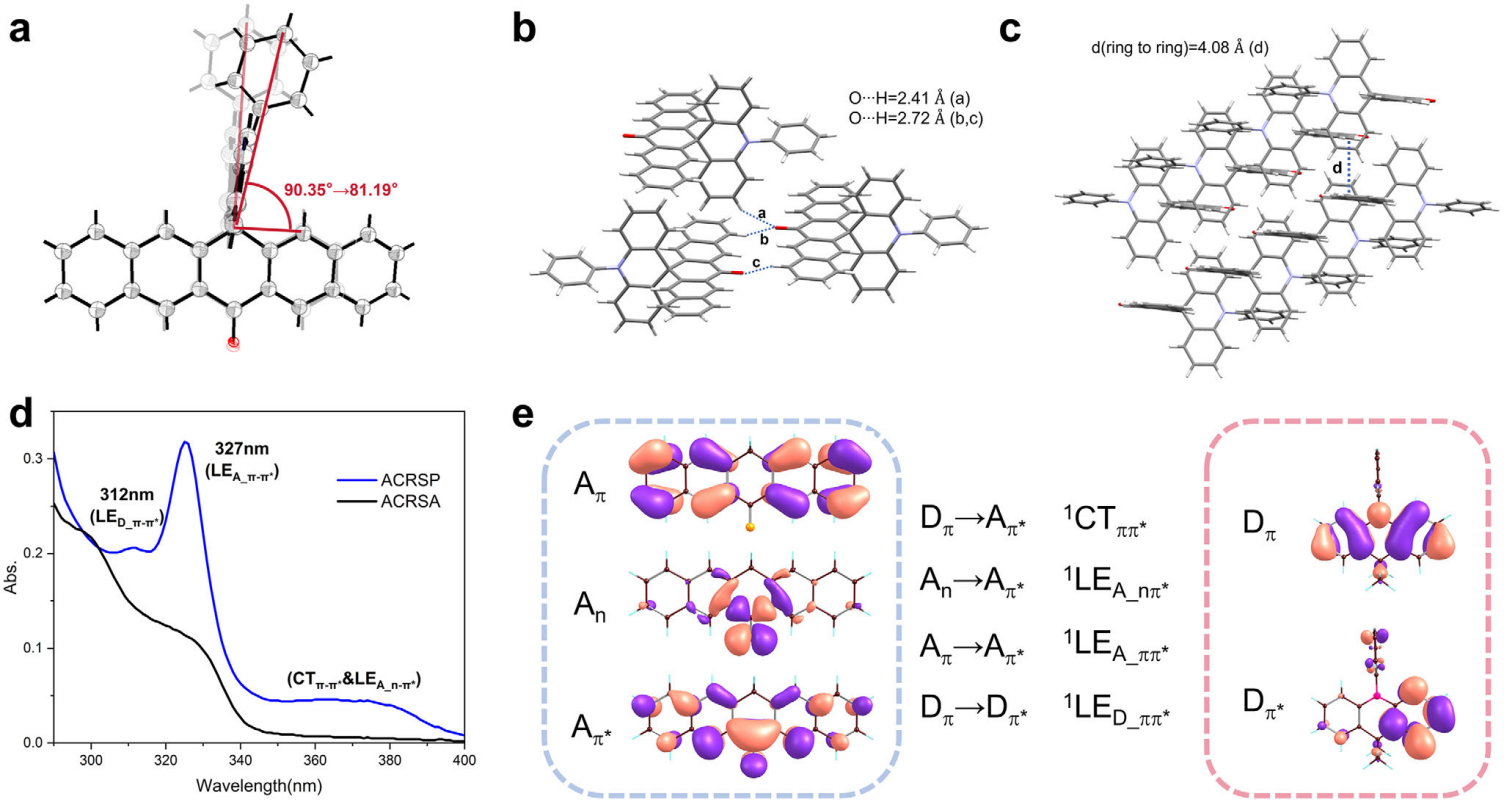
Single‐crystal structure and vertical excitation modes of ACRSP. a–c) Single crystal structure of ACRSP highlighting key bond angles a) hydrogen bonds b) and *π‐π* stacking c). d) Absorption spectra of ACRSA and ACRSP measured at 10 µm concentration in toluene. e) Excitation modes and associated molecular orbitals of ACRSP, represented by donor and acceptor fragments.

The single‐crystal structures reveal notable conformational differences between ACRSP and ACRSA (**Figure** **2**a). ACRSA's dihedral angle between the donor acridine plane and the acceptor anthrone plane is nearly perpendicular. In contrast, ACRSP's larger conjugated area reduces this angle to 81.19°, effectively promoting through‐space CT. The hydrogen bonding formed between the two pentacene ketone units results in an inverted dual parallel stacking (Figure 2b), and the orthogonal structure of ACRSP significantly suppresses intermolecular π‐π stacking (4.08 Å) (Figure 2c).

### 2.2 Vertical Excitation and Transition Modes Analysis

The presence of fused benzene rings on both sides endows ACRSP with significantly stronger absorbance than ACRSA across the 290–400 nm range (Figure 2d). Experiments indicate that the absorption peak of the phenyl acridine unit is ≈287 nm, with absorption ending at 332 nm (Figure S2, Supporting Information). Therefore, for the absorption spectrum after 350 nm in Figure 2d, the signals correspond only to the excitation of the acceptor unit and intramolecular charge transfer (ICT) excitation.

As shown in Figure 2e, theoretical calculations reveal that the peak at 312 nm in ACRSP's absorption spectrum arises from the D_π_→D_π*_ excitation (^1^LE_D_ππ*_) of the phenyl acridine unit, the region ≈327 nm corresponds to the A_π_→A_π*_ transition (^1^LE_A_ππ*_) of the benzene rings in the pentacene ketone unit. The range beyond 350 nm includes both the A_n_→A_π*_ transition (^1^LE_A_nπ*_) from the carbonyl part and the ICT transition (^1^CT_ππ*_). Combined excitation spectra and computational analysis reveal that the ^1^LE_A_nπ*_ transition exhibits higher vertical excitation energy and greater oscillator strength compared to the ICT transition (Table S6, Supporting Information). Together, these features encompass four distinct transition modes: donor π→π* transition, acceptor π→π* transition, acceptor n→π* transition, and ICT transition.

### 2.3 Photophysical Properties of ACRSP in Dilute Solvents

**Figure 3:**
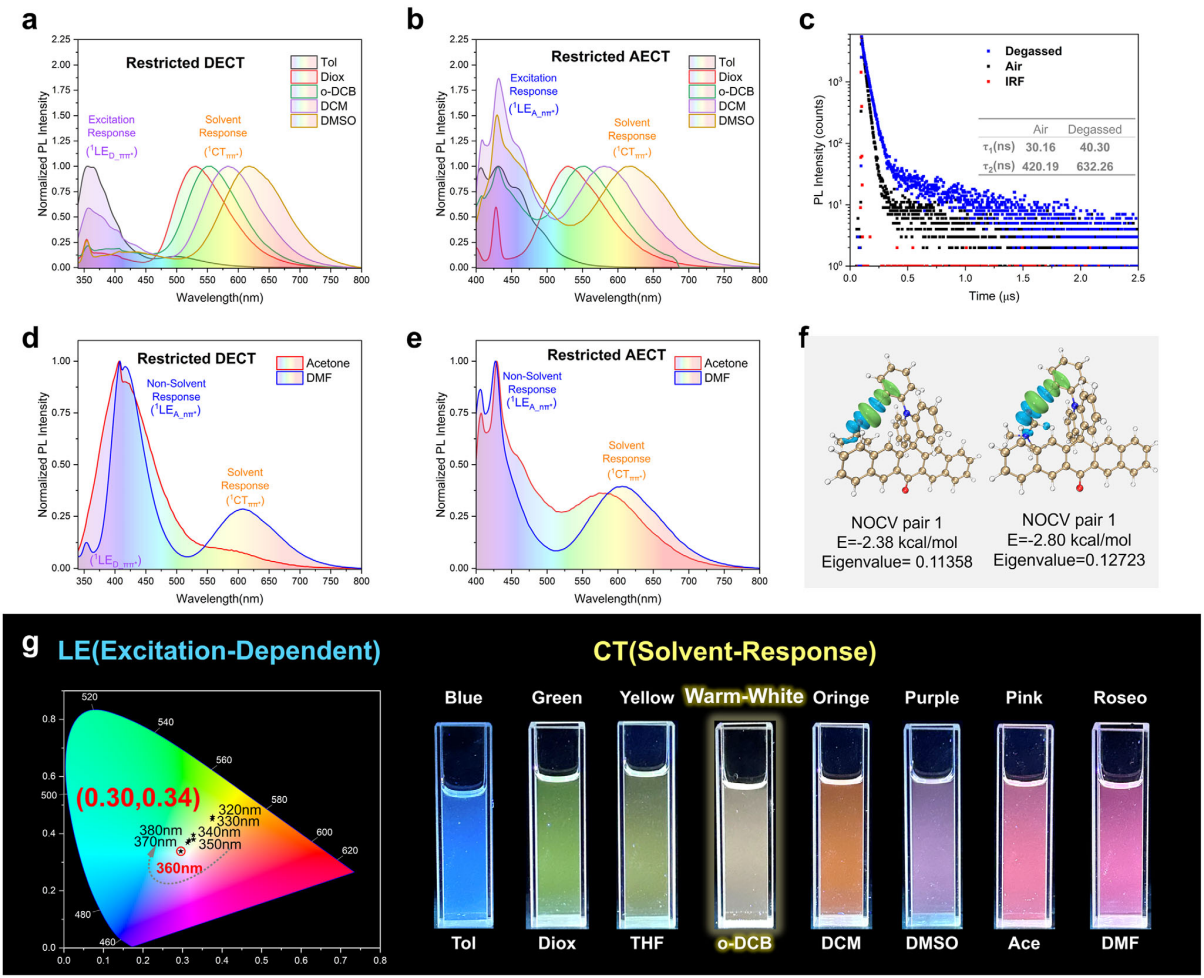
Photophysical properties of ACRSP. a,b) Emission spectra (10 µm) of ACRSP excited at 320nm a) and 380nm b) wavelengths in Tol, Diox, o‐DCB, DCM, DMSO. c) Emission delay curve (10 µm) of the CT emission peak of ACRSP before and after degassing in DMF. d,e) Emission spectra (10 µm) of ACRSP excited at 320nm (d) and 380nm (e) wavelengths in Acetone and DMF. f) The first NOCV pairs of ACRSP in DMF and acetone (with isovalue = 0.0002 a.u.) g) CIE chromaticity diagram of ACRSP at multiple excitation wavelengths in o‐DCB and photos of ACRSP solutions (5 µm) in different solvents under 365 nm irradiation. Tol: toluene, Diox: 1,4‐dioxane, o‐DCB: 1,2‐dichlorobenzene, DCM: dichloromethane, DMSO: dimethyl sulfoxide, DMF: N,N‐Dimethylformamide, Ace: Acetone.

Interestingly, ACRSP exhibits Ex‐De dual fluorescence behavior in dilute solutions of toluene (Tol), 1,4‐dioxane (Diox), 1,2‐dichlorobenzene (o‐DCB), dichloromethane (DCM), and dimethyl sulfoxide (DMSO) (**Figure** **3**a,b). The low‐energy component of the dual fluorescence displays a distinct Gaussian‐shaped emission and solvatochromic effect, which are typical characteristics of the CT state. In contrast, the high‐energy emission peak is independent of solvent polarity, indicative of the LE state.

As the excitation wavelength increases from 320 to 380 nm, the position of the ^1^CT_ππ*_ emission peak remains unchanged, while the LE emission peak shifts from ≈350 to 425 nm. Experiment revealed that the emission peak ≈350 nm corresponds to ^1^LE_D_ππ*_ (Figure S2, Supporting Information). Additionally, the vibrationally coupled emission peaks of ACRSA and ACRSP ≈425 nm align perfectly with the emission spectrum of anthrone (Figure S4, Supporting Information). This indicates that excitation of the donor leads to the coexistence of emission of ^1^LE_D_ππ*_ and ^1^CT_ππ*_ (DECT mode), while the AECT_nπ*_ mode results in ^1^LE_A_nπ*_ fluorescence and ^1^CT_ππ*_ emission. Furthermore, excitation of the ^1^LE_D_ππ*_ state does not produce emission from the ^1^LE_A_nπ*_ state, demonstrating that DECT and AECT_nπ*_ are fundamentally independent (Figure 3a,b).

In fact, the Ex‐De behavior of ACRSP in solution originates from the differences in radiative pathways following local excitation of the donor and acceptor. The spectral independence of ^1^LE_A_nπ*_ and ^1^LE_D_ππ*_ emission bands is induced by the suppression of nonradiative transitions from the LE to the CT state. The calculated ΔE_ST_ of ACRSP is 0.05 eV, indicating the potential for efficient rISC. The lifetime of ^1^CT_ππ*_ emission exhibits distinct biexponential decay dynamics before and after deoxygenation (Figure 3c), Under the condition that the prompt fluorescence component remains almost unchanged, the delayed fluorescence component increases from 420.19 ns (air) to 632.26 ns (degassed), indicating the thermally activated delayed fluorescence (TADF) property of ACRSP.

Surprisingly, carbonyl‐containing solvents exhibit completely different photophysical behavior under DECT. Compared to the distinct Ex‐De dual emission in non‐carbonyl solvents, an additional ^1^LE_A_nπ*_ emission appears in the DECT mode in carbonyl‐containing solvents, while still retaining the ^1^LE_D_ππ*_ state emission (Figure 3d). Significant triple emissions are shown in DMF under 290 and 320 nm excitation (Figure 3d; Figure S22, Supporting Information). The emission spectra under the AECT_nπ*_ mode exhibit similar behavior in both carbonyl‐containing solvents and non‐carbonyl solvents. Analysis of the excitation spectra indicates that the ^1^LE_A_nπ*_ emission primarily originates from direct excitation of the ^1^LE_A_nπ*_ state and excitation of the ^1^LE_D_ππ*_ state (Figure S23, Supporting Information). The clear ^1^LE_A_nπ*_ emission observed under 320 nm excitation is mainly due to the ^1^LE_D_ππ*_ contribution in carbonyl‐containing solvents.

The transition between ^1^LE_D_ππ*_ and ^1^LE_A_nπ*_ involves a two‐electron transfer, and the emission spectrum of the acridine unit overlaps with the absorption spectrum of the pentacene ketone. We speculate that the additional ^1^LE_A_nπ*_ emission in the DECT may follow the Förster resonance energy transfer (FRET) mechanism.^[^ ^17^ ^]^ This hypothesis is supported by the Extended Transition State‐Natural Orbitals for Chemical Valence (ETS‐NOCV) analysis^[^ ^18^ ^]^ performed using the Multiwfn program.^[^ ^19^ ^]^ Additionally, the carbonyl group of DMF can form an orbital interaction of 2.80 kcal mol^−1^ with the phenyl acridine part of ACRSP (Figures S1, S9, and S10, Supporting Information). At the same time, the oxygen atom of the DMF carbonyl group and the carbon and hydrogen atoms on the benzene ring align nearly in a straight line (with a bond angle of 161.42°), which is characteristic of hydrogen bonding. Similar to DMF, acetone also exhibits a hydrogen‐bond‐dominated intermolecular interaction, with 2.35 kcal/mol of orbital interaction.

The calculations of the electronic coupling values for both Dexter energy transfer (DET) and FRET mechanisms in carbonyl‐containing (DMF) and non‐carbonyl (DCM) solvents further elucidate the differences in photophysical behavior of ACRSP (Table S1, Supporting Information). Using a mixed‐solvent model (an implicit solvent model combined with one explicit solvent molecule) to account for solvent‐induced changes. When DCM is used as the solvent, the FRET coupling value is 2.81 × 10^−7^ hartree^2^, which is comparable to the DET pathway value of 4.76 × 10^−7^ hartree^2^. However, when DMF is used, the FRET coupling increases by an order of magnitude to 1.67 × 10^−6^ hartree^2^, indicating that the carbonyl‐containing solvent DMF can significantly promote intramolecular FRET between the donor and acceptor in the ACRSP molecule.

Intermolecular hydrogen bonding alters the orthogonal structure of ACRSP by enhancing the orbital coupling between the donor and acceptor, significantly reducing the spatial distance between the acridine donor unit and the pentacene ketone acceptor unit, thereby facilitating FRET from the donor to the acceptor. This further confirms that the separation of DECT and AECT_nπ*_ excitation modes originates from the unique rigid orthogonal structure of ACRSP, and this property responds to the carbonyl group in the environment.

The concentration‐dependent excitation spectra of the CT emission peak directly reveal the different spectral dynamics arising from changes in excitation modes (Figures S24 and S25, Supporting Information). Three characteristic peaks originate from different excitation modes: the DECT contribution gradually decreases in the 310–320 nm range, while the AECT_ππ*_ contribution continuously increases in the 326–340 nm range, and the AECT_nπ*_ (mixed ICT) region shows a significant enhancement near 378 nm. Both DECT and AECT require the formation of an LE state as a prerequisite for CT. With increasing concentration, high‐energy states transfer energy to lower‐energy states via FRET, reducing the stability of LE states and thereby diminishing their contribution to CT. Excitation‐mode separation enables the Ex‐De multi‐emission of ACRSP to be concentration‐responsive. The increase in concentration causes the emission peak to shift toward a single wavelength, indicating that the anti‐Kasha emission is an intrinsic isolated molecule behavior rather than an effect of molecular aggregation.

Interestingly, the LE emission of ACRSP in solution exhibits Ex‐De characteristics, while the solvent polarity only affects CT fluorescence (Figure 3g). This unique stimulus‐responsive behavior enables programmable control of multi‐emission to achieve more complex composite‐color luminescence. Compared to the ultraviolet emission from the ^1^LE_D_ππ*_ state of ACRSP, the ^1^LE_A_nπ*_ emission peak of pentacene ketone is located in the blue‐light region, making it an ideal blue component for white‐light illumination. Meanwhile, the CT emission in o‐dichlorobenzene solution appears in the complementary yellow‐light region. At an excitation wavelength of 360 nm, ACRSP emits white light with CIE coordinates of (0.30, 0.34) (Figure 3g; Figure S26, Supporting Information), demonstrating the effectiveness of excitation‐mode separation in achieving tunable white‐light emission.

### 2.4 Formation and Relaxation of Triplet Excitons

In fact, investigating the generation and relaxation of triplet excitons is crucial for introducing RTP into Ex‐De systems. The TADF properties of ACRSP indicate that the relaxation pathway of the excited state must involve triplet states. According to El‐Sayed rule,^[^ ^20^ ^]^ rapid ISC is more favorable in the pentacene ketone unit containing the n‐π* state. However, the nearly degenerate energy levels of ^1^LE_A_nπ*_ and CT make internal conversion (IC) a strong competitor to ISC. Therefore, a theoretical model is urgently needed to accurately describe the triplet‐state photophysical behavior of ACRSP.

Due to the complex nonadiabatic and avoided crossings involved in the anti‐Kasha multi‐level emission and Ex‐De processes of ACRSP, multi‐reference methods like N‐Electron Valence State Perturbation Theory (NEVPT2)^[^ ^21^ ^]^ are more advantageous than single‐reference methods (such as DFT) for investigating the thermodynamic changes of excited states during electron transfer.^[^ ^22^ ^]^ Marcus‐Levich‐Jortner (MLJ) theory^[^ ^23^ ^]^ has been proven to be an effective model for quantitatively evaluating the influence of IC and triplet‐state transitions on excited‐state dynamics.^[^ ^24^ ^]^ Therefore, we employed NEVPT2(10e/10o)/def2‐TZVP^[^ ^25^ ^]^ within the MLJ theoretical framework to assess the thermodynamics and kinetics of ACRSP, aiming to establish a viable theoretical model for the triplet exciton properties in ACRSP. Detailed theoretical and computational procedures are provided in the Supporting Information.

**Figure 4:**
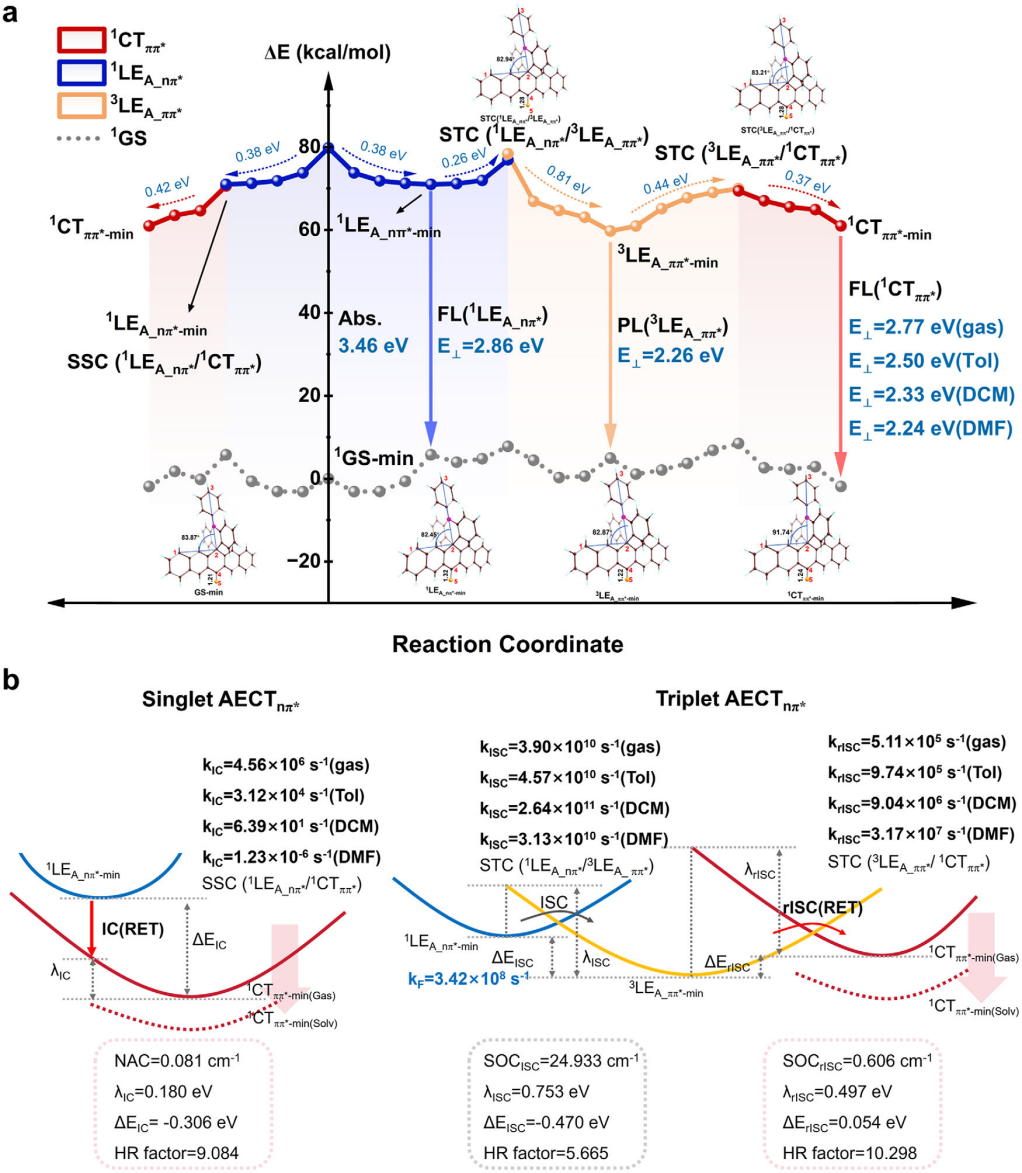
Thermodynamics and kinetics analyses of the AECT_nπ*_ pathway. a) The minimum energy profile (MEP) of ACRSP for the AECT_nπ*_ processes, the structural evolution along the relaxation path, and its key bond parameters are provided. b) The calculated non‐radiative rate constants for AECT_nπ*_ were shown in both singlet and triplet states, along with the corresponding electronic coupling values (NAC and SOC), reorganization energy (λ), electronic energy gap (ΔE), and Huang‐Rhys (HR) factor, within the framework of the MLJ theory. The computational results were obtained at the NEVPT2/def2‐TZVP//IRC/CASSCF(10e/10o) level in gas and solvents. SSC: Singlet‐Singlet Crossing, STC: Singlet‐Triplet Crossing, and RET: Restricted Electron Transfer.

With the solvent polarity increasing, the CT energy level decreases (from 2.77 to 2.24 eV) (**Figure** **4**a), transitioning the rISC process from endothermic to exothermic. In DMF, the rISC rate reaches 3.17 × 10^7^ s^−1^ (Figure 4b), matching the radiative rate of ^1^LE_A_nπ*_. In ACRSP, the exciton recombination (fluorescence) rate of ^1^LE_A_nπ*_ is comparable to the rISC rate, resulting in dual‐level emission. Due to the strong polarity of DMF, the larger endothermic process and weaker electronic coupling both hinder ISC from ^1^CT_ππ*_ to ^3^LE_A_ππ*_, making its rate significantly lower than the ISC rate from ^1^LE_A_nπ*_ to ^3^LE_A_ππ*_ (Table S6, Supporting Information). Therefore, we infer that the delayed CT component in polar solvents is more likely contributed by ^1^LE_A_nπ*_. The solvent effect has less impact on the LE state, causing the ISC process between ^1^LE_A_nπ*_ and ^3^LE_A_ππ*_ to be scarcely affected, with the rate varying within two orders of magnitude and being much faster than the rISC rate.

The *π‐π* stacking interaction between DA fragments causes the ground‐state minimum structure of ACRSP to collapse from a C2v symmetry to near Cs symmetry along the plane of the pentacene ketone, with the donor‐acceptor bond angle σ_∠1‐2‐3_ is 83.87°. The thermodynamic changes along the relaxation pathways and the key bond parameters are shown in Figure 4a. Upon photoexcitation to the Frank‐Condon (FC) region of the ^1^LE_A_nπ*_ state, ACRSP rapidly relaxes to the ^1^LE_A_nπ*‐min_ state, accompanied by an exothermic release of 0.38 eV. During this process, the carbonyl bond lengthens from 1.21 to 1.32 Å. At this point, either singlet or triplet AECT_nπ*_ processes may occur, with the triplet pathway discussed first. ACRSP overcomes a 0.26 eV barrier via STC (^1^LE_A_nπ*_/^3^LE_A_ππ*_), causing the carbonyl bond to initially shorten to 1.28 Å. Kinetic calculations reveal a strong SOC of 24.933 cm^−1^, enabling an ISC rate of 3.90 × 10^10^ s^−1^ (gas) between the ^1^LE_A_nπ*_ and ^3^LE_A_ππ*_ states (Figure 4b). The rapid ISC process drives a large number of excitons into the ^3^LE_A_ππ*_ state. Subsequently, the molecule relaxes to the ^3^LE_A_ππ*‐min_ state, accompanied by further carbonyl shortening to 1.22 Å and releasing 0.81 eV of heat. The small ΔE_rISC_ allows excitons to reach the ^1^CT_ππ*_ state via an endothermic pathway involving 0.44 eV (in the gas phase) through STC (^3^LE_A_ππ*_/^1^CT_ππ*_). At this stage, the rISC rate is calculated as 5.11 × 10^5^ s^−1^ (gas). Caused by long‐range electron transfer (ET), rISC is identified throughout the process as the rate‐determining step for triplet AECT_nπ*_.

The AECT_nπ*_ pathway in the singlet state requires ACRSP to reach the ^1^CT_ππ*_ state by the avoided crossing point SSC (^1^LE_A_nπ*_/^1^CT_ππ*_). Due to minimal overlap between the acridine orbital and the carbonyl n orbital, the transition is difficult, resulting in the NAC of only 0.081 cm^−1^ between the ^1^LE_A_nπ*_ and ^1^CT_ππ*_ states. The IC rate between these two states is 4.56 × 10^6^ s^−1^, significantly lower than the rISC rate between the ^3^LE_A_ππ*_ and ^1^CT_ππ*_ states. Moreover, as the solvent polarity increases, the energy gap between the ^1^LE_A_nπ*_ and ^1^CT_ππ*_ states widens, further reducing the IC rate by more than ten orders of magnitude (Table S7, Supporting Information).

This indicates that when the ^1^LE_A_nπ*_ state in ACRSP serves as the precursor, it reaches the ^1^CT_ππ*_ state via a triplet‐state‐mediated pathway, and the SOC of up to 24.933 cm^−1^ makes this process more favorable than accessing the triplet state from the ICT. Despite their close energy levels, the extremely small NAC of 0.081 cm^−1^ still leads to a forbidden IC between the ^1^LE_A_nπ*_ and ^1^CT_ππ*_ states. Therefore, AECT_nπ*_ and ICT are essentially two independent excitation modes that generate different exciton spin distributions.

### 2.5 Photophysical Properties of ACRSP in Rigid Matrix

**Figure 5:**
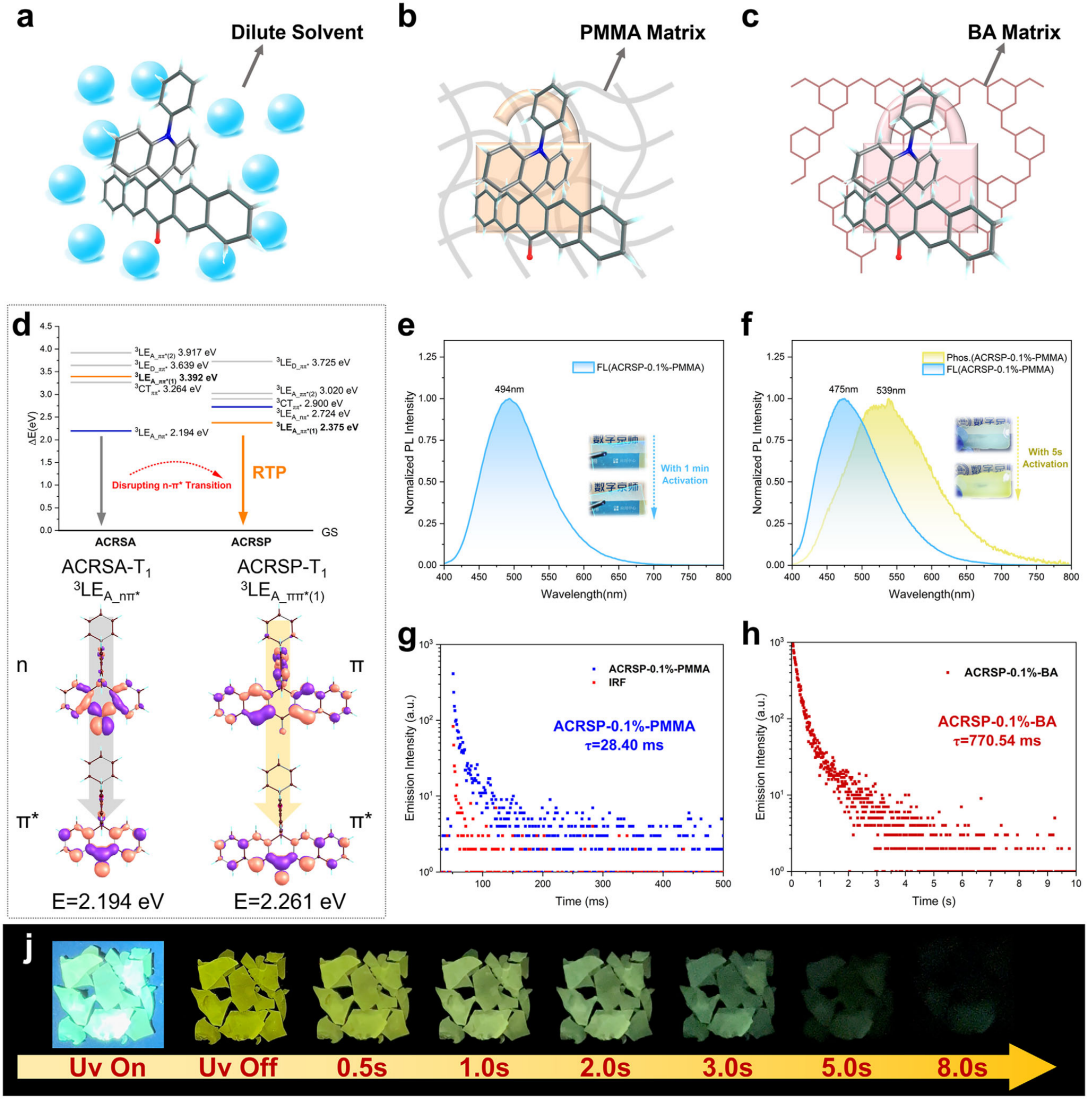
Photophysical properties of ACRSP doped in PMMA and BA matrix. a–c) Schematic diagram of the single‐molecule behavior of ACRSP under solvent host a), PMMA host b), and BA matrix host c). d) Triple state energy level and transition property of ACRSA and ACRSP at T_1_ state minimum point calculated at the NEVPT2/def2‐TZVP//CASSCF(10e/10o) level. e) Fluorescence spectra of ACRSA‐0.1%‐PMMA film at 365 nm excitation. f) Phosphorescence and fluorescence spectra of ACRSP‐0.1%‐PMMA film at 365 nm excitation, with 1 ms delay for phosphorescence. g) The emission delay curve of the RTP of ACRSP‐0.1%‐PMMA. h) The emission delay curve of the RTP of ACRSP‐0.1%‐BA. i) Photos of ACRSP‐0.1%‐BA before and after turning off the UV 365 nm light.

In dilute solution, thermal dissipation induced by molecular vibration prevents RTP with low radiative rates from occurring (**Figure** **5**a). Triplet excitons are rapidly converted into TADF emission through rISC. In the rigid matrix, the environment formed by covalent bonds, van der Waals forces, hydrogen bonding, and π‐π interactions suppresses nonradiative triplet energy loss caused by molecular vibrations while also preventing oxygen‐induced triplet quenching (Figure 5b,c). Due to its high transparency and ease of processing, poly(methyl methacrylate) (PMMA) was chosen as a rigid network to restrict the non‐radiative transitions of ACRSP. Importantly, PMMA has relatively low polarity, which increases the system's ΔE_ST_ to limit rISC (Figure 4b). The ACRSA‐0.1%‐PMMA and ACRSP‐0.1%‐PMMA films were prepared by the solution‐casting method, exhibiting colorlessness, flexibility, and high transparency. Under 365 nm excitation, ACRSA‐0.1%‐PMMA and ACRSP‐0.1%‐PMMA exhibit sky‐blue ^1^CT_ππ*_ fluorescence emissions at 494 nm and 475 nm, respectively (Figure 5d,e). Notably, ACRSP‐0.1%‐PMMA undergoes significant photochromism after brief photoactivation, changing from sky blue to yellow‐green (Figure 5e). Upon removal of the excitation source, the film emits a visually perceptible yellow afterglow (τ = 28.40 ms), demonstrating its unique RTP properties (Figure 5g). This photoactivation phenomenon was related to oxygen in the polymer. In contrast, ACRSA‐0.1%‐PMMA retains its sky‐blue emission even after 1 min of continuous irradiation, without any phosphorescence observed. The afterglow emission of ACRSP‐0.1%‐PMMA at 544 nm closely matches the phosphorescence of ACRSP in o‐DCB solution at 77 K (541 nm), confirming that the rigid covalent polymer network suppresses vibrational motion and facilitates T_1_ state emission (Figure S27, Supporting Information). The photoactivation characteristics of RTP allow us to record information repetitively through light or use it as an anticounterfeiting method.

Theoretical calculations indicate that the phosphorescence difference between ACRSP and ACRSA originates from the transition of the T_1_ state composition (Figure 5f). Similar to benzophenone, the lowest triplet n‐π* state in ACRSA features near‐unity intersystem crossing (ISC) efficiency but exhibits a short phosphorescence lifetime.^[^ ^15^ ^]^ By fusing benzene rings at both ends of the acceptor, the high ISC rate is maintained while the triplet n‐π* state at T_1_ is disrupted. The inverted energy levels enhance the orbital overlap, favoring phosphorescent emission, enabling ACRSP to exhibit millisecond‐scale afterglow emission under ambient conditions.

When ACRSP was dispersed into a dehydrated boric acid (BA) matrix using the melt‐casting method, impressively, the ultralong organic RTP lifetime of ACRSP‐0.1%‐BA was significantly extended to 770.54 ms (Figure 5h), with afterglow persisting for up to 5 s (Figure 5i). The extensive triangular BO_3_ units in boron oxide (B_2_O_3_) form a cross‐linked network, providing greater rigidity compared to linear polymers, thereby suppressing non‐radiative transitions.^[^ ^10^ ^]^ On the other hand, RTP materials are highly sensitive to air and water. Compared to the solution‐casting method, the melt‐casting system contains less water, preventing the quenching of triplet excitons due to environmental factors. The anchoring interactions between fluorophores and supramolecular structures play a crucial role in RTP systems. Although inorganic oxides can exhibit excellent phosphorescent properties, their intrinsic rigidity often limits their processability for flexible anti‐counterfeiting applications. In contrast, polymer films such as PMMA, with their outstanding flexibility, optical transparency, and ease of fabrication, provide a more practical and versatile platform for exploring excitation‐responsive luminescent materials.

**Figure 6:**
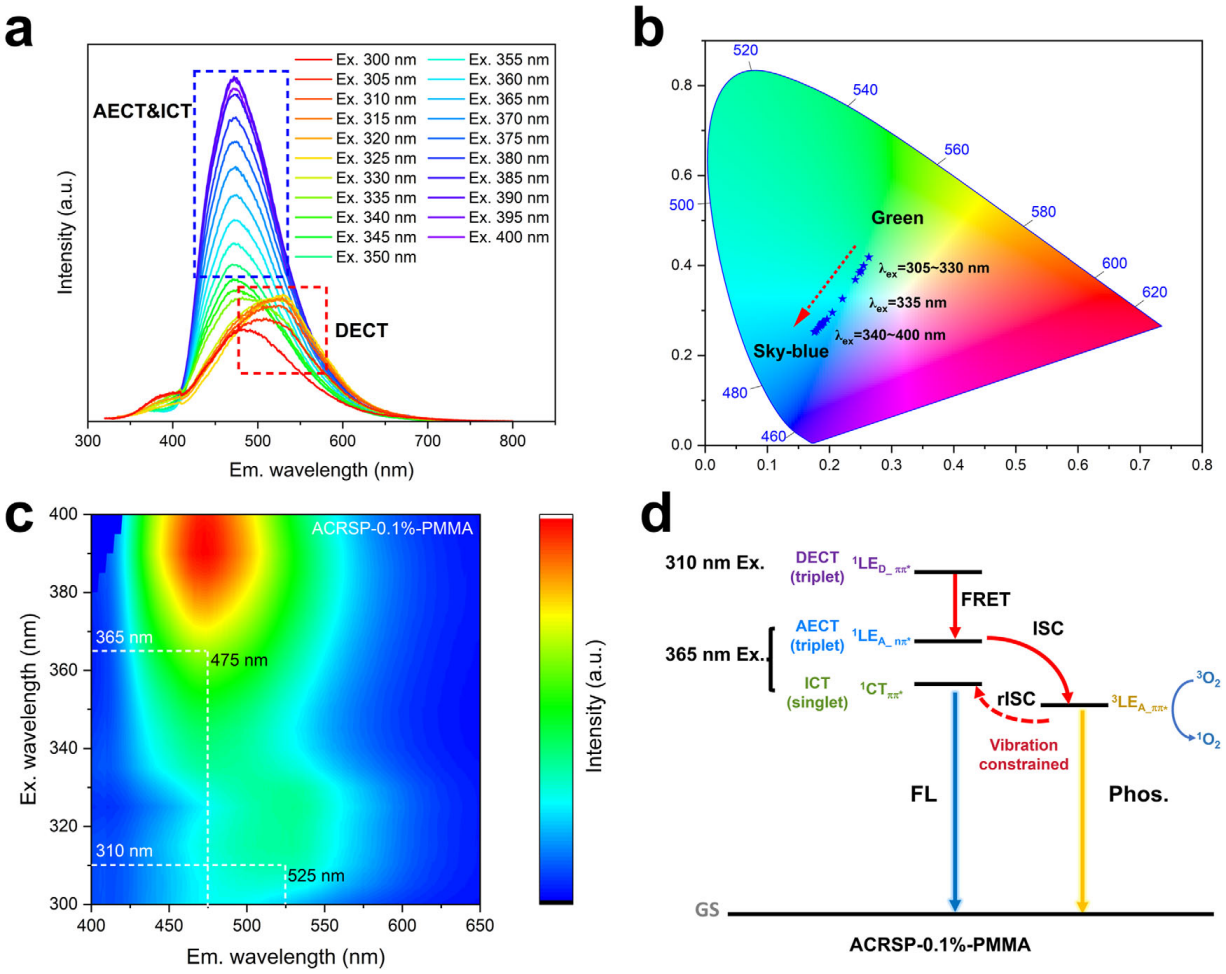
Excitation‐dependent properties of the ACRSP‐0.1%‐PMMA. a–c) Luminescence spectra a) contour map b) and CIE chromaticity diagram c) of ACRSA‐0.1%‐PMMA. d, Proposed mechanism of ACRSP under PMMA host.

To investigate the impact of excitation modes on luminophores in the polymer matrix, the multi‐excitation wavelength scan was performed on ACRSP‐0.1%‐PMMA. Surprisingly, unlike in dilute solutions where the low‐energy peaks are unaffected by the excitation wavelength, ACRSP‐0.1%‐PMMA shows opposite Ex‐De behavior, with low‐energy peaks responding to the excitation wavelength and high‐energy peaks remaining unchanged (**Figure** **6**a). Under excitation wavelengths of 305–330 nm, the emission peak redshifted to 525 nm (green), whereas increasing the excitation wavelength to 340–380 nm resulted in a blueshift back to 475 nm (sky‐blue) (Figure 6b; Figure S28, Supporting Information). In the heat map, the excitation‐dependent spectral regions displayed a distinct separation boundary, which closely aligned with the absorption cutoff of the acridine unit (332 nm). This corresponds well with the boundary between the DECT and AECT&ICT excitation modes (Figure 6c). The ^1^LE_A_nπ*_ emissions induced by anti‐Kasha behavior are still preserved in ACRSP‐0.1%‐PMMA. Interestingly, the ^1^LE_A_nπ*_ emission peak appears only when the excitation wavelength is below 340 nm and becomes almost indistinguishable beyond 340 nm. The absorption of ^1^LE_A_nπ*_ is highly dependent on the Herzberg‐Teller (HT) effect.^[^ ^26^ ^]^ After vibrational restriction, the absorption intensity of the ^1^LE_A_nπ*_ level is significantly reduced, making ICT the dominant transition mode under low‐energy excitation. Moreover, due to the presence of abundant hydrogen bond acceptors (ester groups) in the PMMA network, the FRET pathway between the ^1^LE_D_ππ*_ and ^1^LE_A_nπ*_ energy levels is activated, similar to the effect observed in carbonyl‐containing solvents (Figure 3d). The additional ^1^LE_A_nπ*_ peak observed under high‐energy excitation further supports this pathway. In this case, the DECT mode undergoes FRET to reach ^1^LE_A_nπ*_, followed by rapid ISC to the triplet state, generating phosphorescent components. Compared to the fast ISC induced at the ^1^LE_A_nπ*_ level, the ISC from the ^1^CT_ππ*_ level to ^3^LE_A_ππ*_ is slower, resulting in fewer triplet excitons, which makes fluorescence emission more favorable under low‐energy excitation (Figure 6d). As the excitation wavelength increases, the proportion of the phosphorescent component gradually decreases, causing ACRSP‐0.1%‐PMMA to exhibit a 50 nm emission wavelength shift in dual‐mode excitation and transition from green to sky‐blue emission. After photoactivation, different excitation modes can induce a strong yellow afterglow, achieving the synergistic integration of Ex‐De and RTP under a single‐emitter system.

### 2.6 Applications for Information Encryption and Dynamic Printing

**Figure 7:**
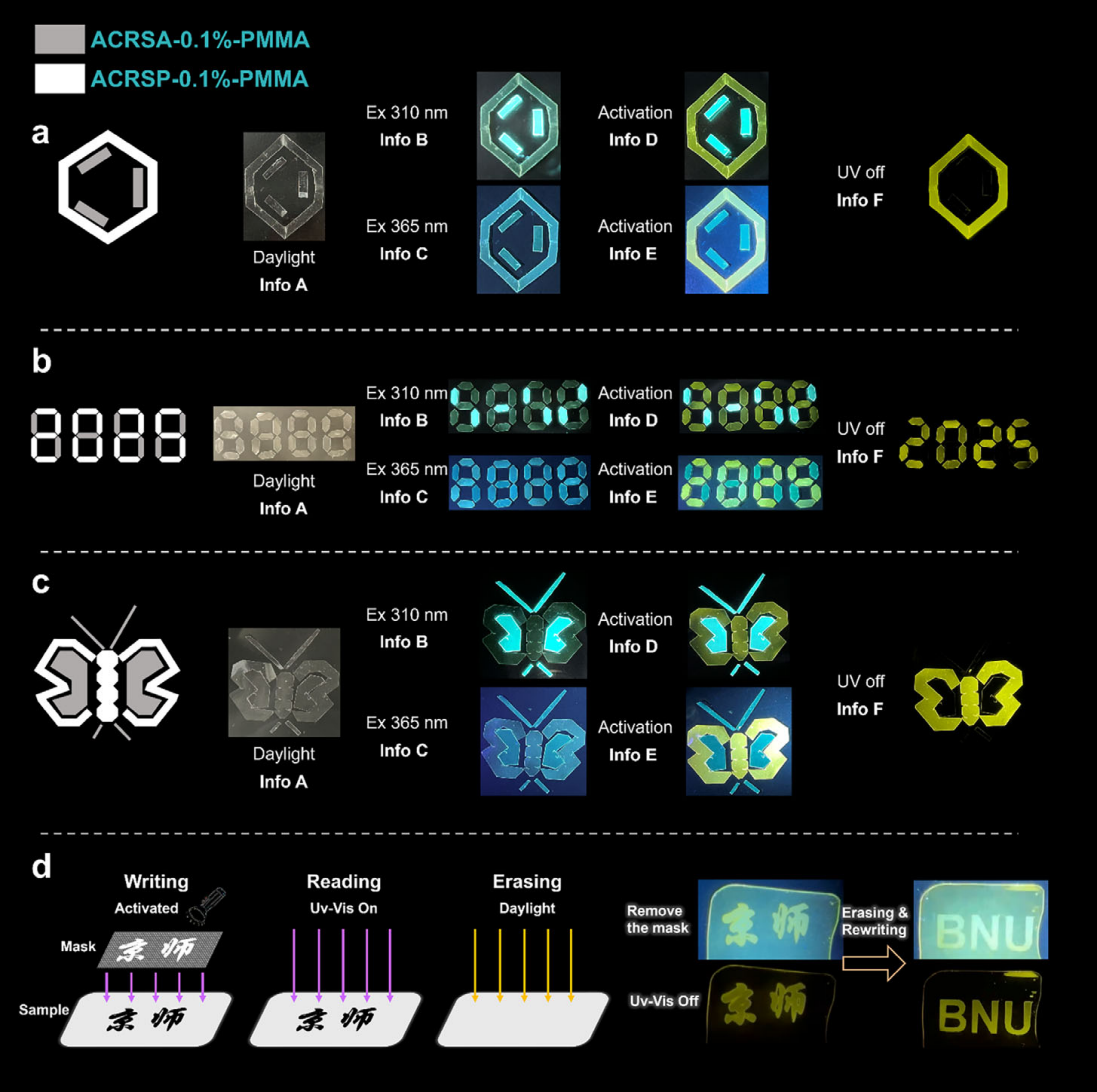
Multi‐dimensional codes for advanced dynamic information encryptions by ACRSP‐0.1%‐PMMA and ACRSA‐0.1%‐PMMA. a–c). Photos of chemical structure, numbers, and butterfly image by ACRSP‐0.1%‐PMMA and ACRSA‐0.1%‐PMMA under daylight, 310 nm, and 365 nm UV light before and after activation, and after removal of the UV light. d, Schematic diagram of mask printing project and multilayer programmable printing based on ACRSP‐0.1%‐PMMA.

By leveraging the complex Ex‐De emission and RTP characteristics of ACRSP, we demonstrated its unique excitation‐responsive and time‐dependent logical variability, providing significant insights for advanced multi‐level programmable logic information encryption (**Figure** **7**a–c). We designed various patterns and encrypted digits for wavelength detection, using ACRSA as a camouflage pattern. Under daylight, the anti‐counterfeiting patterns appear colorless and transparent, making them indistinguishable (Info A). When exposed to 365 nm UV light, the visually indistinguishable blue emission may convey misleading information (Info B). However, under 310 nm UV irradiation, ACRSP‐0.1%‐PMMA exhibits a PL shift from 475 nm to 525 nm, while ACRSA‐0.1%‐PMMA only shows enhanced brightness (Info C). After photoactivation, ACRSP‐0.1%‐PMMA transitions to yellow emission, and upon 365 nm excitation, it presents a dual‐color pattern with similar brightness (Info D). Under 310 nm excitation, the camouflage pattern is highlighted (Info E). Finally, after the light source is removed, the system transitions to a sixth‐level encoded state with only yellow afterglow emission (Info F). To expand its applications, this material was used in the write‐read‐erase process for a programmable tag (Figure 7d). With patterned illumination, any design can serve as a template for phosphorescent information storage. For example, the “京师” (Beijing Normal University) mask was placed on an ACRSP‐0.1%‐PMMA film and illuminated with 365 nm UV light for 5 s. After removing the mask, no visible changes occurred under ambient light. However, upon re‐illumination with UV light, the doped film displayed phosphorescent “京师” characters against a background of sky‐blue fluorescence, indicating successful information storage. This generated image remained activated for tens of seconds, and when the excitation source was turned off, the background darkened while the “京师” characters emitted a yellow afterglow. After 5 min in natural conditions, the information self‐erased. Repeating the process with a “BNU” mask allowed easy updating of the stored information, demonstrating the versatility of this material for reprogrammable information encryption applications.

## 3 Conclusion

In this work, we report a general emission strategy that integrates Ex‐De emission and RTP through donor‐acceptor excitation‐mode separation. This strategy enables complex luminescence behavior and multi‐level information encryption under ambient conditions in a rigid, heavy‐atom‐free, isolated molecular system. The approach relies on decoupling emission pathways under different excitation modes. Experimental and theoretical results demonstrate that ACRSP exhibits distinct Ex‐De afterglow emissions in flexible and rigid matrices, with up to four energy levels participating cooperatively. The triplet‐dominated AECT_nπ*_ pathway is key to the multi‐emission Ex‐De behavior. In dilute solution, Ex‐De behavior is restricted to high‐energy LE states, and the combination with solvent‐responsive CT states allows programmable white‐light emission control. In contrast, in the rigid matrix, the disruption of the triplet n‐π* excited state collectively induces a transition from TADF to ultralong organic RTP, and changes in triplet components give rise to Ex‐De behavior from low‐energy RTP emissions. Owing to ACRSP's unique excitation‐responsiveness and time‐dependent logic variability, it has been applied in multi‐level information encryption and dynamic printing. This work expands the conventional model of anti‐Kasha emission in isolated molecules and provides both theoretical and experimental insights for achieving novel multi‐level information encryption under complex environments.

## Conflict of Interest

The authors declare no conflict of interest.

## Supporting information



Supporting Information

## Data Availability

The data that support the findings of this study are available in the supplementary material of this article.
