# Supplementary material for: Excitation‐Dependent Quadruple‐Level Emission from an Isolated Molecule for Dynamic Information Encryption
Source: Adv Sci (Weinh). 2025 Jul 11;12(38):e08987. doi: 10.1002/advs.202508987 (PMC12520544; doi:10.1002/advs.202508987)
Supplement: Supplementary file 1 — Supporting Information [file ADVS-12-e08987-s001.pdf]

ADVANCED SCIENCE
Open Access

Supporting Information

for *Adv. Sci.*, DOI 10.1002/advs.202508987

Excitation-Dependent Quadruple-Level Emission from an Isolated Molecule for Dynamic Information Encryption

Yibo Shi, Lin Liu, Wei-Hai Fang, Qian Wang, Xiao Liu, Kai Feng, Wei Sun, Dongpeng Yan and Xuebo Chen**

Supporting Information

Excitation-Dependent Quadruple-Level Emission From an Isolated Molecule for Dynamic Information Encryption

Yibo Shi[a] #, Lin Liu[a] #, Wei-Hai Fang[a], Qian Wang[a], Xiao Liu[a], Kai Feng[a], Wei Sun[a], Dongpeng Yan*[a], Xuebo Chen*[a] [b]

Table of Contents

1. Theory and calculations. 1

2. Supplementary data of Phenyl Acridine, Pentaphenylketone, Anthrone, ACRSA and ACRSP. 9

3. Materials and methods 13

4. Instrumentation and analytical techniques 14

5. Synthetic procedures 14

6. X-ray crystallography. 16

7. ^{1}H, ^{13}C NMR and ESI-MS spectra. 17

8. Photophysical properties of ACRSP 22

9. The critical points for the minimum energy profile (MEP) of NEVPT2 // CASSCF calculations 25

10. Reference. 43

1. Theory and calculations.

DFT Methods: All density functional theory (DFT) and time-dependent density functional theory (TD-DFT) calculations for the ACRSP molecule were performed at the PBE0(D3BJ)/def2-SVP[26, 28] level. Geometry optimizations for both ground and excited states were carried out using the single-crystal diffraction structure as the initial guess. Vibrational analyses were subsequently conducted to confirm that the optimized structures correspond to minima without any imaginary frequencies, and the resulting Hessian matrix was used for further analysis. The Franck-Condon (FC) approximation assumes that electronic transitions are much faster than molecular vibrations, so nuclear displacement can be neglected during the transition. However, the FC approximation does not account for transitions where the transition dipole moment becomes non-zero due to molecular vibrations, leading to an underestimation of oscillator strength. To accurately describe transitions forbidden by symmetry that become allowed due to vibrationally induced changes in the dipole moment, the Herzberg-Teller (HT) effect must be considered[27]. The HT effect modifies the transition matrix elements by introducing a first-order Taylor expansion of the dipole moment with respect to vibrational modes, enabling transitions such as the $n \rightarrow \pi^*$ transition of carbonyl groups, which are typically forbidden under the Franck-Condon approximation. The Tamm-Dancoff approximation (TDA) within time-dependent DFT was employed to evaluate the influence of the HT effect on oscillator strengths and the related dynamics. Based on the DFT-derived Hessian matrix, the $n \rightarrow \pi^*$ state oscillator strengths for absorption and emission were corrected for HT effects using the TDA-PBE0(D3BJ)/def2-SVP level. This part of DFT calculations was conducted using the ORCA 5.0.3 software package[29].

The extended transition state-natural orbitals for chemical valence (ETS-NOCV) method[19] were used to analyze weak interactions between solvent molecules and ACRSP because it allows for a detailed decomposition of the orbital interaction energy into contributions from individual NOCV pairs, providing insights into the energy and electron density changes associated with different types of orbital interactions between fragments. The complex between ACRSP and an explicit solvent molecule was considered, and the complex's conformation was optimized at the PBE0(D3BJ)/def2-SVP/IEFPCM[26, 28, 30] level, with vibrational analysis confirming that the obtained structure has no imaginary frequencies. DFT calculations for the mixed solvent model were performed using Gaussian 16 A.03[31], and the ETS-NOCV analysis was calculated by Multiwfn 3.8 and VMD programs[20, 32].

CASSCF and NEVPT2 Methods: Single-reference methods assume that the electronic configuration of a system is dominated by a single leading configuration. However, at conical intersections or avoided crossings, multiple electronic configurations contribute almost equally, and single-reference methods fail to capture such complex behaviors. At the potential energy surface (PES) crossing points, both the electronic and geometric structures of molecules can undergo significant changes, making simple single-reference methods, such as Hartree-Fock or DFT calculations, insufficiently accurate. In such cases, the coupling between multiple electronic states and pronounced electron correlation effects necessitate the use of multi-reference methods, such as multi-reference configuration interaction (MRCI) or complete active space self-consistent field (CASSCF), which are more suited for addressing these complexities[23a, 33]. N-electron valence state perturbation theory (NEVPT2) avoids the intruder-state problem without requiring additional empirical correction parameters, offering significant advantages for high-accuracy multireference state calculations. The initial guess structures for multireference calculations were derived from the stationary points on the single-reference potential energy surface and optimized geometries using state-averaged CASSCF calculations over ten equally weighted roots. Vertical excitation energies were calculated at the fully internally contracted NEVPT2 (FIC-NEVPT2) level across ten roots to account for both dynamic and static correlation effects. The NEVPT2 calculations utilized Ahlrichs' def2-TZVP basis set for all C, H, O, and N atoms, with the resolution of identity (RI) technique employed to accelerate the Coulomb and exchange term calculations[34]. The conductor-like polarizable continuum model (C-PCM) is used to correct the vertical excitation energy at the NEVPT2 level to account for the influence of solvent on the potential energy surface[35]. Multireference methods are highly sensitive to the selection of the active space, making them more complex compared to single-reference approaches. To ensure accurate results for ACRSP, the active space was carefully determined using a fragment-based approach. Large space multireference calculations were performed separately for the donor (phenyl acridine) and acceptor (pentacene ketone) fragments to identify the key orbitals involved in excitation. This step enabled the selection of an appropriate active space for ACRSP that balances computational cost and accuracy. The final active space included 10 electrons in 10 orbitals, ensuring a reliable description of photophysical relaxation processes within practical computational limits. Geometry optimizations were performed using OpenMolcas v24.02[36], and all vertical excitation energy calculations were performed with ORCA 5.0.3[29]. The transfer of molecular orbitals between different programs is assisted by the MOKIT package[37].

To study conical intersections (CI), we employed a state-averaged CASSCF

approach with ten equally weighted states to optimize the PES at the crossing points. This method ensures a balanced and accurate treatment of electronic correlation across multiple states by averaging the energies of these states. During the search for conical intersections, we focused on two types of crossing points: singlet-triplet crossing (STC) and singlet-singlet crossing (SSC). To optimize the geometric structures of these intersections, we adopted the minimum energy crossing point (MECP) method. This technique is commonly used for optimizing the structures of regions where non-adiabatic coupling between electronic states occurs, aiming to identify the lowest-energy structures near the crossings. For optimizing STC points, the energy optimization was performed under the constraint that the energy difference between the singlet and triplet states remains zero. This ensures that during optimization, the energies of the two different spin states are strictly equal, enabling accurate localization of the crossing point. Similarly, SSC points were optimized under the same constraint, ensuring the energy difference between the two singlet states was zero. Based on the MECP structures, non-adiabatic coupling (NAC) between singlet states was calculated using the ALASKA module. For spin-orbit coupling (SOC) between singlet and triplet states, we employed the full-electron atomic mean-field integrals (AMFI) algorithm. Accurate SOC calculations directly influence the transition rates between singlet and triplet states, impacting the prediction of molecular photophysical behavior and dynamic evaluations. ΔE_{ST} was obtained by projecting onto the ground-state geometry in the gas phase.

Analysis of rate constants: Marcus theory has been successfully applied to the calculation of non-radiation relaxation. Marcus posited that the energy scale of electronic vibrational transitions is much smaller than that of any other relevant energy scale in the system. In this scenario, the expression for the electron transfer rate is as follows[24b, 24c]:

$$k_{Marcus} = \frac{2\pi|H_{ab}|^2}{\hbar\sqrt{4\pi\lambda k_B T}} \exp\left(-\frac{(\Delta G^0+\lambda)^2}{4\lambda k_B T}\right) \quad (1)$$

Where H_{ab} represents the electronic coupling from the initial state to the final state. T denotes temperature. $\hbar$ stands for the reduced Planck constant. k_B is the Boltzmann constant, λ is the reorganization energy, and ΔG^0 is the Gibbs free energy difference between the relaxed structure of the initial and final electronic states.

Jortner introduced quantized molecular vibrations into Marcus theory, accounting for the quantum effects of high-frequency vibrational modes[24a]. Each mode is characterized by a Huang-Rhys factor (S_j) and a frequency (ω_j). The vibrational modes

can be represented as a series of energy levels, with spacings equal to the quantum vibrational energy ($\hbar\omega$). The Franck Condon weighted density gradually converges as the vibrational quantum number(n) increases. n is taken as 100 in this work. The full active space (FAS) MLJ rate expression is:

$$k_{MLJ-FAS} = \frac{2\pi}{\hbar}|H_{ab}|^2\sqrt{\frac{1}{4\pi\lambda k_B T}}\exp\left(\sum_{j=1}^{N} -S_j\right)\sum_{n_1=0}^{\infty}\cdots\sum_{n_2=0}^{\infty}\cdots\sum_{n_N=0}^{\infty}\times\left(\prod_{j=1}^{n}\frac{{S_j}^{n_j}}{n_j!}\right)$$

$$\exp(-\frac{(\lambda+\sum_{j=1}^{N} n\hbar\omega_{eff}+\Delta G^0)^2}{4\lambda k_B T}) \qquad (2)$$

Calculations based on the full active space equation are highly time-consuming, with computational costs escalating rapidly for larger molecules. However, studies have shown that approximating with one effective mode provides excellent accuracy. The one effective mode MLJ equation is as follows:

$$k_{MLJ-eff} = \frac{2\pi}{\hbar}|H_{ab}|^2\sqrt{\frac{1}{4\pi\lambda k_B T}}\sum_{n=0}^{+\infty}\frac{e^{-S}S^n}{n!}\exp\left(-\frac{(\lambda+n\hbar\omega_{eff}+\Delta G^0)^2}{4\lambda k_B T}\right) \qquad (3)$$

Where ω_{eff} is calculated based on the Huang-Rhys (HR) factor as:

$$\omega_{eff} = \frac{\sum_{j=1}^{\infty} S_j\omega_j}{S} \qquad (4)$$

Fermi's Golden Rule can be applied because the states are only weakly coupled[38]. However, Marcus electron transfer theory underestimates the rate constant values for molecules in the inverted region. The Marcus-Levich-Jortner (MLJ) theory overcomes this issue by incorporating corrections to the density of state factors to account for the electron-vibrational coupling between the states involved in the transition[24a]. This represents a major advancement in the field, as it is the first rigorously derived method capable of describing nuclear quantum effects in electron transfer reactions, remaining effective across nearly the entire temperature range. The MLJ theory has been widely used for predicting and interpreting non-radiative rates, to guide the synthesis and rational design of high-performance semiconductors applicable to organic photoluminescence. The Huang-Rhys factor in the formula can be calculated by projecting the reaction coordinate vector onto the displacement vector of normal mode coordinates, utilizing the FCHT module in Gaussian 16 A.03[31]. Several studies have shown that, particularly for the Marcus normal and inverted regions, only values of $S_j \geq 0.03$ are considered representative of vibrational modes[25]. We have adopted the same approach in this study.

To accurately represent the donor-localized excitation energy levels ($^1LE_{D\_\pi\pi^*}$),

acceptor-localized excitation energy levels ($^1LE_{A\_n\pi^*}$ and $^1LE_{A\_\pi\pi^*}$), charge-transfer excitation energy level ($^1CT_{\pi\pi^*}$), and phosphorescent emission energy level ($^3LE_{A\_\pi\pi^*}$), the active space for ACRSP was ultimately determined to be (10e, 10o). This active space includes the carbonyl n orbital, π/π* orbitals from two acridine units, and two bonding π orbitals along with three π* orbitals of the pentacene ketone unit. This selection sufficiently captures the electronic correlation effects and potential energy surface crossings associated with ACRSP's multiple charge-transfer emission modes. The potential energy surface profile is corrected for energy using the fully internally contracted N-Electron valence state perturbation theory (FIC-NEVPT2) level. All vertical excitation energy calculations were performed with ORCA 5.0.3[29].

Analysis of Energy Tranfer: According to Fermi's golden rule, the transition rate is determined by the square of the transition matrix element and the overlap between the initial and final state wavefunctions (the Franck–Condon factor).

$$W_{i\to f} = \frac{2\pi}{\hbar} \cdot |\langle \Phi_i|\hat{H}'|\Phi_f\rangle|^2 \cdot \sum_{u,v} P_{iu}\, |\langle \Theta_{iu} \mid \Theta_{fv} \rangle|^2 \delta(E_{fv} - E_{iu}) \qquad (5)$$

where Φ and Θ represent electronic and vibrational wavefunctions, respectively, i and f denote the initial and final adiabatic electronic states, respectively, u and v denote nuclear vibrational states corresponding to electronic states i and f, respectively, $\hat{H}'$ is the nonadiabatic transition operator to perturb the system from state i to f, E_{iu} and E_{fv} are energies of vibronic states, and P_{iu} is the Boltzmann factor. The nuclear part in Eq (1), which is so-called as the Franck-Condon (FC) term, can be calculated based on the multidimensional harmonic oscillator model as

$$\sum_{u,v} P_{iu}\, |\langle \Theta_{iu} \mid \Theta_{fv} \rangle|^2 \delta(E_{fv} - E_{iu}) = \frac{1}{2\pi\hbar} \int dt e^{it\omega_{i\to f}} \prod_j G_j(t) G_j(t) =$$

$$\sum_{u_j,v_j} P_{iu_j} \left| \left\langle \chi_{iu_j} \mid \chi_{fv_j} \right\rangle \right|^2 e^{it\left(v_j+\frac{1}{2}\right)\omega_j} e^{-it\left(u_j+\frac{1}{2}\right)\omega_j} \qquad (6)$$

Here χ denotes the nuclear wavefunction of harmonic oscillators, ω_j is the vibrational frequency of the j_{th} normal mode, and $\omega_{i\to f}$ is the adiabatic energy difference between electronic states i and f. The energy levels of the energy transfer donor acridine unit and the energy transfer acceptor pentacene ketone unit are not affected by the solvent. The conformational changes of the initial and final states remain the same under different solvent environments. Therefore, the Franck–Condon (FC) factor remains unchanged, and the variation in energy transfer rates across different solvents arises from differences in the square of the transition dipole matrix element.

The definition of transition dipole moment is different in Dexter energy transfer

(DET) and FRET. The DET mechanism assumes that the two electrons are localized on the donor and acceptor, respectively. Upon interaction between an excited donor molecule (D*) and a ground-state acceptor (A), the system transitions to a ground-state donor (D) and an excited acceptor (A*), involving the simultaneous transfer of two electrons. This process entails wavefunction exchange and reflects the nature of "electron exchange," which is characteristic of Dexter transfer, requiring orbital overlap and antisymmetry of the wavefunction. The transition dipole moment can be expressed as follows:

$$\left|\langle \Phi_i | \hat{H}' | \Phi_f \rangle\right|^2_{DET} = \langle \varphi_{D*}(1)\varphi_A(2) | \frac{1}{r_{12}} | \varphi_{A*}(1)\varphi_D(2)\rangle^2 =$$

$$\sum_{i,j,k,l} c_i(\varphi_{D*}) c_j(\varphi_{A*}) c_k(\varphi_A) c_l(\varphi_D)(ij \mid kl) \qquad (7)$$

Where φ_{D*}, φ_D, φ_{A*} and φ_A are singly occupied orbitals of donor (D) and acceptor (A), respectively, 1 and 2 denote the two exchanged electrons during energy transfer, (ij|kl) denotes a two-electron integral with (i, j) and (k, l) as the basis sets associated with electron 1 and 2, respectively, and c is the coefficient of the singly occupied orbitals.

FRET does not involve the intermolecular physical transfer of electrons but rather the resonant transfer of excited-state energy, which is primarily governed by electrostatic dipole–dipole interactions. As such, the wavefunctions of the two molecules can be considered localized, with an electron relaxing from D* to D while simultaneously another electron is excited from A to A*, without any spatial exchange between them. For the FRET process, the electronic part is often approximated as classical dipole–dipole interactions between the donor and acceptor.

However, in our system, the center-to-center distance between the energy-transfer donor acridine unit and the acceptor pentacene ketone unit is 3.05 Å, at which point the influence of electronic structure on FRET becomes dominant. To more accurately describe the transition dipole moment of FRET at such a short distance, we adopted Eq. 6 proposed by Lin et al. for our calculations[39].

$$\left|\langle \Phi_i | \hat{H}' | \Phi_f \rangle\right|^2_{FRET} = \langle \varphi_{D*}(1)\varphi_A(2) | \frac{1}{r_{12}} | \varphi_D(1)\varphi_{A*}(2)\rangle^2 =$$

$$\sum_{i,j,k,l} c_i(\varphi_{D*}) c_j(\varphi_D) c_k(\varphi_A) c_l(\varphi_{A*})(ij \mid kl) \qquad (8)$$

Table S1. Difference in energy transfer coupling values of ACRSP under two different mixed solvents (implicit solvent model combined with one explicit solvent molecule).

	E_{donor} (kcal/mol)	$E_{accrptor}$ (kcal/mol)	$\left|\langle\Phi_i|\hat{H}'|\Phi_f\rangle\right|^2$ (hartree^2)
ACRSP_DCM_FRET	80.78	70.26	2.81×10^{-7}
ACRSP_DCM_DET	80.78	70.26	4.76×10^{-7}
**ACRSP_DMF_FRET**	**80.78**	**70.26**	$\mathbf{1.67\times10^{-6}}$
ACRSP_DMF_DET	80.78	70.26	7.89×10^{-7}

Two key factors governing energy transfer are the energy levels of the donor and acceptor, and the extent of orbital coupling. Since the energy levels of $^1LE_{D\_\pi\pi^*}$ and $^1LE_{A\_n\pi^*}$ states are not influenced by solvent polarity, the additional $^1LE_{A\_n\pi^*}$ emission observed under the DECT mode can only originate from the effect of carbonyl-containing solvents on orbital coupling.

Whether FRET can occur in non-carbonyl solvents is a critical issue. We have supplemented our study with the calculated energy transfer coupling values for both DET and FRET in carbonyl-containing (DMF) and non-carbonyl (DCM) solvents. Using a mixed-solvent model (implicit solvent model combined with one explicit solvent molecule) to account for solvent-induced changes, we found that when DCM is used as the solvent, the FRET coupling value is 2.81×10^{-7} hartree^2 (Table S1), which is comparable to the DET pathway value of 4.76×10^{-7} hartree^2. However, when DMF is used, the FRET coupling increases by an order of magnitude to 1.67×10^{-6} hartree^2, indicating that the carbonyl-containing solvent DMF can significantly promote intramolecular FRET between the donor and acceptor in the ACRSP molecule.

Analysis of hydrogen bond: To verify the presence of hydrogen bonding, the key atomic charges and bond parameters for both carbonyl and non-carbonyl solvents have been calculated. As shown in Figure S1, the ∠C–H···O angle between ACRSP and DMF is 161.42°, with a key O···H hydrogen bond (HB) length of 2.30 Å. In contrast, in DCM, the ∠C–H···O decreases to 128.9°, and the O···H hydrogen bond length increases to 3.10 Å.

Since hydrogen bonding is primarily governed by polarization and electrostatic interactions[40], Mulliken charge analysis indicates that the oxygen atoms in DMF and acetone carry charges of -0.362 and -0.267, respectively, which are significantly more negative than the -0.134 carried by the chlorine atom in DCM. The greater negative charge enhances polarization and electrostatic attraction, forming the intrinsic basis for

hydrogen bond formation between carbonyl solvents and ACRSP.

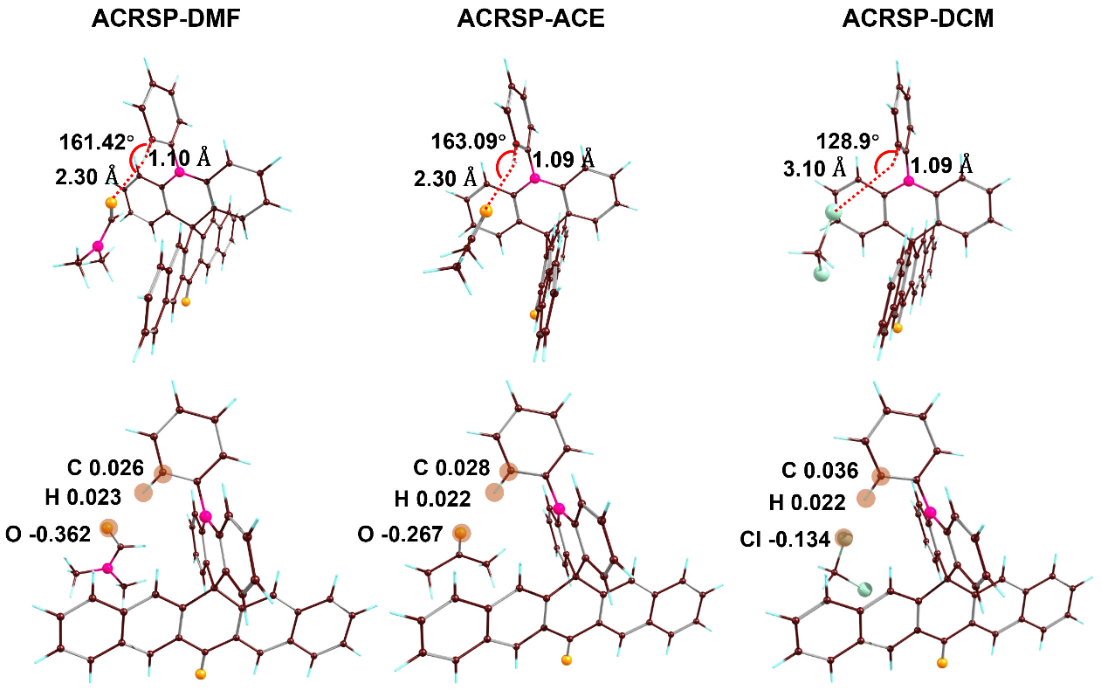

Figure S1. The hydrogen bond parameters and key atomic Mulliken charges formed between ACRSP molecules and explicit solvent molecules (DMF, acetone and DCM)

Table S2. The electron density at the bond critical point of hydrogen bonding between ACRSP and solvent molecules, and the predicted hydrogen bonding energies.

	DMF	ACE	DCM
ρ(BCP)/a.u.	0.0144	0.0128	0.0042
E(HB)/kcal·mol-1	-2.47005	-2.11312	-0.1947

Since hydrogen bond strength is directly related to the properties at the bond critical point (BCP), such as electron density, Laplacian of the electron density, energy density, and potential energy density[41], we evaluated the hydrogen bond strength between ACRSP and the explicit solvent molecules using the empirical formula proposed by Lu et al.[42].

$$E(HB) = -223.08 * \rho(BCP) + 0.7423 \qquad (9)$$

DMF and acetone can form weak hydrogen bonds with ACRSP, with binding strengths of 2.47 kcal/mol and 2.11 kcal/mol (Table S2), respectively, demonstrating the unique advantage of carbonyl-containing solvents in hydrogen bond formation. In contrast, due to weaker polarization and electrostatic interactions, DCM forms a much

weaker hydrogen bond with ACRSP (−0.19 kcal/mol), making it less effective in influencing the photophysical properties.

2. Supplementary data of Phenyl Acridine, Pentaphenylketone, Anthrone, ACRSA and ACRSP.

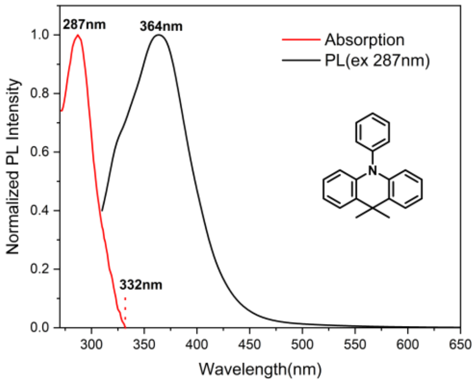

Figure S2. Experiment absorption and emission spectra of the phenyl acridine in toluene (10 μM).

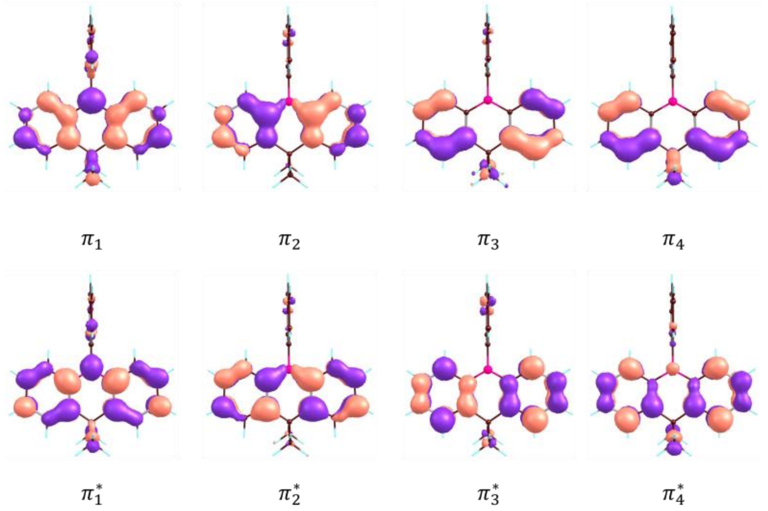

Figure S3. Molecular orbitals of phenyl acridine used in defining the active space for the NEVPT2 / def2-TZVP // CASSCF(8e/8o) calculations.

Table S3. Mainly transitions, vertical excitation energies (E⊥, eV), oscillator strengths (f), wavelength(nm), and singly occupied molecular orbitals (SOMOs) of phenyl acridine. These values were calculated at the 10-root NEVPT2 / def2-TZVP // CASSCF(8e/8o) / level of theory.

Transitions	$E_\perp$(eV)	f	Wavelength(nm)	SOMO
Absorbtion				
$S_0 \rightarrow {}^1LE_{D\_\pi\pi^*(1)}$	4.089	0.327837025	303.2	$\pi_1 \rightarrow \pi_2^*$
$S_0 \rightarrow {}^1LE_{D\_\pi\pi^*(2)}$	4.368	0.024721814	283.9	$\pi_1 \rightarrow \pi_4^*$
$S_0 \rightarrow {}^1LE_{D\_\pi\pi^*(3)}$	4.687	0.040942798	264.5	$\pi_1 \rightarrow \pi_3^*$
$S_0 \rightarrow {}^1LE_{D\_\pi\pi^*(4)}$	5.371	0.092130561	230.9	$\pi_4 \rightarrow \pi_2^*$
$S_0 \rightarrow {}^1LE_{D\_\pi\pi^*(5)}$	5.550	0.005292733	223.4	$\pi_4 \rightarrow \pi_4^*$
Emission				
${}^1LE_{D\_\pi\pi^*(1)} \rightarrow S_0$	3.725	0.272783335	332.8	$\pi_1 \rightarrow \pi_2^*$

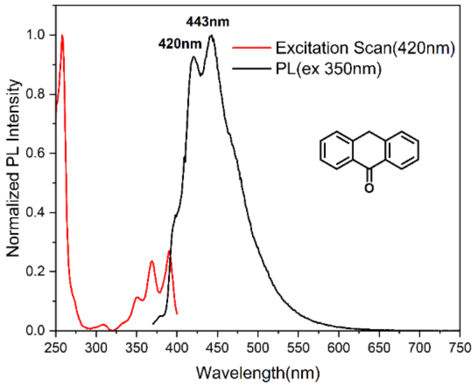

Figure S4. Experiment excitation and emission spectra of the anthrone in PMMA (1 wt%).

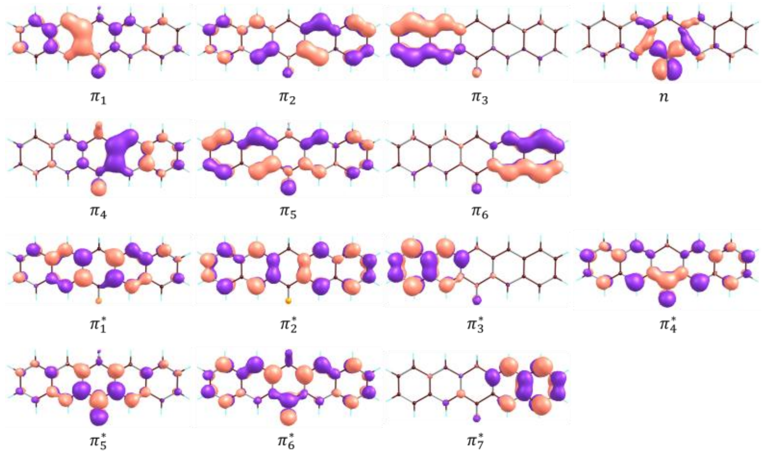

Figure S5. Molecular orbitals of pentaphenylketone used in defining the active space for the NEVPT2 / def2-TZVP //CASSCF(14e/14o)/def2-TZVP calculations.

Table S4. Mainly transitions, vertical excitation energies ($E_\perp$, eV), oscillator strengths (f), wavelength(nm), and singly occupied molecular orbitals (SOMOs) of pentaphenylketone. These values were calculated at the 10-root NEVPT2 / def2-TZVP

// CASSCF (14e/14o) level of theory.

Transitions	E⊥(eV)	f	Wavelength(nm)	SOMO
		Absorbtion		
$S_0 \rightarrow {}^1LE_{A\_n\pi^*}$	3.570	0.000113257	347.3	$n \rightarrow \pi_1^*$
$S_0 \rightarrow {}^1LE_{A\_\pi\pi^*(1)}$	4.265	0.032237963	290.7	$\pi_5 \rightarrow \pi_4^*$
$S_0 \rightarrow {}^1LE_{A\_\pi\pi^*(2)}$	4.419	0.528522747	280.5	$\pi_2 \rightarrow \pi_4^*$
$S_0 \rightarrow {}^1LE_{A\_\pi\pi^*(3)}$	4.554	0.056792292	290.7	$\pi_1 \rightarrow \pi_4^*$
$S_0 \rightarrow {}^1LE_{A\_\pi\pi^*(4)}$	4.746	0.047628530	280.5	$\pi_4 \rightarrow \pi_4^*$
		Emission		
${}^1LE_{A\_n\pi^*} \rightarrow S_0$	2.756	0.000003184	449.8	$n \rightarrow \pi_4^*$

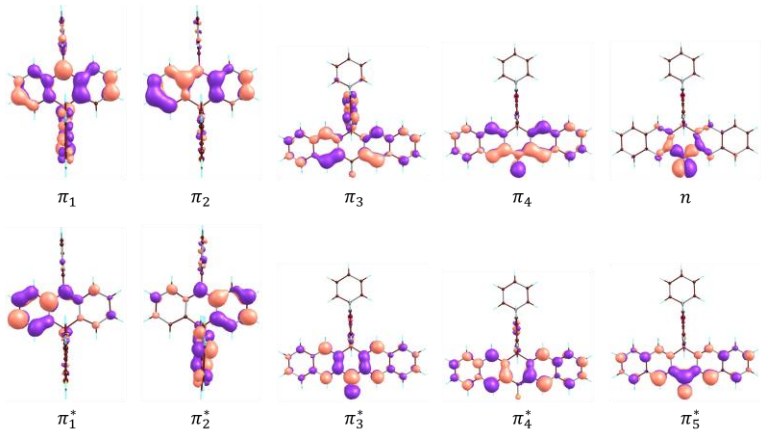

Figure S6. Molecular orbitals of **ACRSP** used in defining the active space for the NEVPT2//CASSCF(10e/10o) calculations.

Table S5. Mainly transitions, vertical excitation energies (E⊥, eV), oscillator strengths (f), wavelength(nm), and singly occupied molecular orbitals (SOMOs) of **ACRSP**. Absorbtion values were calculated at the 15-root NEVPT2 / def2-TZVP / /CASSCF(10e/10o) level of theory. Emission values were calculated at the 10-root NEVPT2 / def2-TZVP //CASSCF(10e/10o) level of theory.

Transitions	E⊥(eV)	f	Wavelength(nm)	SOMO
		Absorbtion		
$S_0 \rightarrow {}^1CT_{\pi\pi^*}$	3.125	0.002052963	396.7	$\pi_1 \rightarrow \pi_5^*$
$S_0 \rightarrow {}^1LE_{A\_n\pi^*}$	3.237	0.000002845	383.0	$n \rightarrow \pi_5^*$
$S_0 \rightarrow {}^1LE_{A\_\pi\pi^*(1)}$	3.758	0.304226480	329.9	$\pi_3 \rightarrow \pi_5^*$
$S_0 \rightarrow {}^1LE_{D\_\pi\pi^*(1)}$	3.782	0.250354478	327.8	$\pi_1 \rightarrow \pi_1^*$
$S_0 \rightarrow {}^1LE_{A\_\pi\pi^*(2)}$	3.826	0.179448443	324.1	$\pi_4 \rightarrow \pi_5^*$

$S_0 \rightarrow {}^1LE_{D\_\pi\pi^*(2)}$	4.316	0.221808927	287.2	$\pi_1 \rightarrow \pi_2^*$
Emission				
${}^1CT_{\pi\pi^*} \rightarrow S_0$	2.725	0.000006262	455.5	$\pi_1 \rightarrow \pi_5^*$
${}^1LE_{A\_n\pi^*} \rightarrow S_0$	2.828	0.000010533	438.4	$n \rightarrow \pi_5^*$

Table S6. Mainly transitions, oscillator strengths (f), the proportion of Herzberg-Teller effect (HT%) and singly occupied molecular orbitals (SOMOs) of **ACRSP**. Absorbtion values were calculated at the TDA-PBE0/def2-SVP under the HT effect.

Transitions	f	HT%.	SOMO
$S_0 \rightarrow {}^1CT_{\pi\pi^*}$	0.000591184	74.26%	$\pi_1 \rightarrow \pi_5^*$
$S_0 \rightarrow {}^1LE_{A\_n\pi^*}$	0.011546688	98.31%	$n \rightarrow \pi_5^*$

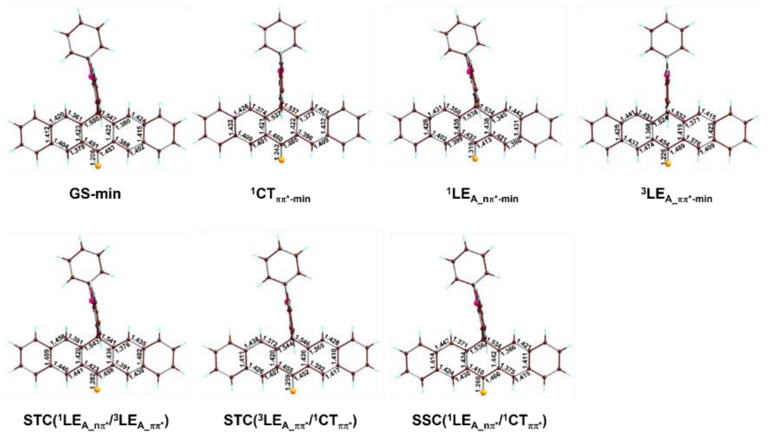

Figure S7. Key bond lengths (Å) involved in potential energy surface minima and crossing points during the $AECT_{n\pi^*}$ process of **ACRSP**.

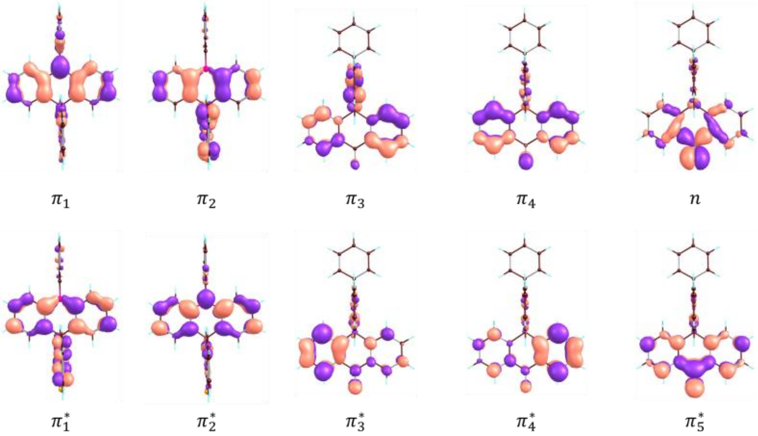

Figure S8. Molecular orbitals of **ACRSA** used in defining the active space for the NEVPT2 / def2-TZVP //CASSCF(10e/10o) calculations.

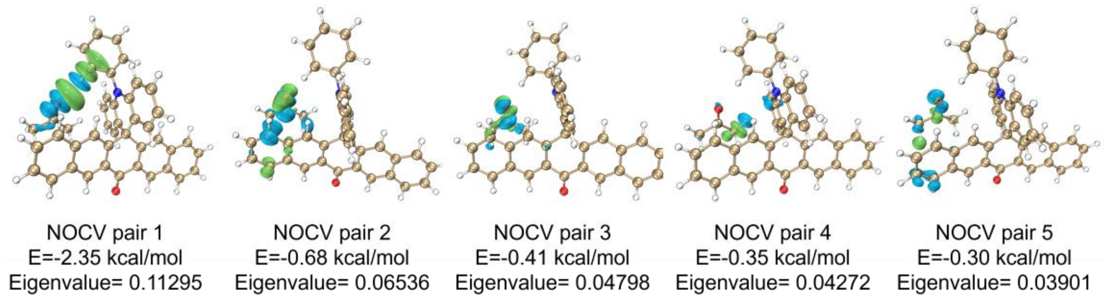

Figure S9. The first five NOCV pairs of **ACRSP** in acetone (with isovalue=0.0002 a.u.).

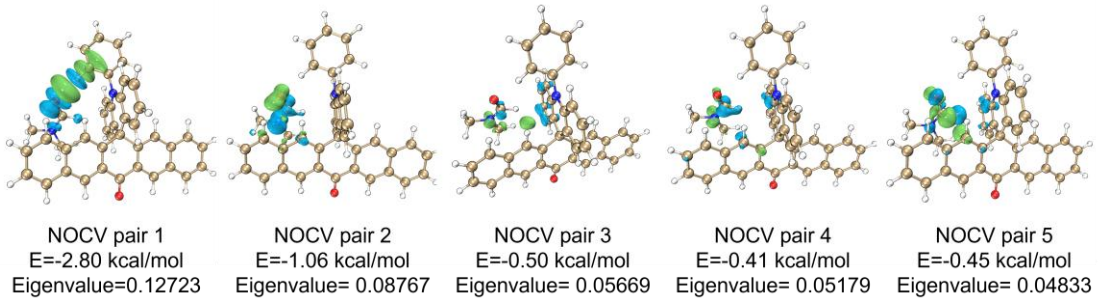

Figure S10. The first five NOCV pairs of **ACRSP** in DMF (with isovalue=0.0002 a.u.).

Table S7. Key non-radiative rates in the AECT$_{n\pi*}$ pathway of **ACRSP**, calculated at the NEVPT2 / def2-TZVP //CASSCF(10e/10o) level (with non-radiative rates in s^{-1}).

Solv.	ISC ($^1CT_{\pi\pi*}\rightarrow{}^3LE_{A\_\pi\pi*}$)	rISC ($^3LE_{A\_\pi\pi*}\rightarrow{}^1CT_{\pi\pi*}$)	ISC ($^1LE_{A\_n\pi*}\rightarrow{}^3LE_{A\_\pi\pi*}$)	rISC ($^3LE_{A\_\pi\pi*}\rightarrow{}^1LE_{A\_n\pi*}$)	IC ($^1LE_{A\_n\pi*}\rightarrow{}^1CT_{\pi\pi*}$)
Gas	4.97×10^{6}	5.11×10^{5}	3.90×10^{10}	1.29×10^{1}	4.56×10^{6}
Tol	4.67×10^{4}	9.74×10^{5}	4.57×10^{10}	6.97×10^{-6}	3.12×10^{4}
DCM	6.94×10^{2}	9.04×10^{6}	2.64×10^{11}	6.97×10^{-16}	6.39×10^{1}
DMF	3.25×10^{-1}	3.17×10^{7}	3.13×10^{10}	1.76×10^{-7}	1.23×10^{-6}

3. Materials and methods

All starting chemicals were obtained from commercial sources and used without further purification unless otherwise specified. All reactions were performed in dried glassware. Anhydrous tetrahydrofuran was obtained from a solvent purification system. All reactions were carried out under a nitrogen atmosphere using standard Schlenk techniques. Thin films were prepared by dissolving the resulting ACRSP and polymethyl methacrylate (PMMA) mixture in chloroform at room temperature, followed by solvent evaporation at room temperature for 12 hours to yield transparent

ACRSP-0.1%-PMMA films. The doped film was pre-photoactivated for 20 seconds prior to photophysical measurements. Other doped films were prepared using the same method. The boric acid (BA) doped material was prepared via a dehydration condensation method. First, a water/1,4-dioxane (1/1, v/v) solution containing ACRSP and BA was evaporated to ensure uniform distribution of the dopant throughout the mixture. The resulting precursor powder was then heated at 150 °C for 20 minutes to achieve complete dehydration and obtain the final material.

4. Instrumentation and analytical techniques

NMR spectra were obtained with a JEOL Delta (600 MHz) using chloroform-d ($CDCl_3$) as solvents. The chemical shifts were confirmed relative to the standard solvent signals reported in the literature. The reference standards are as follows: the chemical shift of $CDCl_3$ in the ^{1}H NMR spectrum is 7.26 ppm, and in the ^{13}C NMR spectrum, it is 77.16 ppm. High-resolution mass spectrometry (HRMS) measurements were conducted using an AB SCIEX Triple TOF 5600+ electrospray ionization-high resolution mass spectrometer (ESI-HRMS). UV-Vis absorption spectra were recorded using a Shimadzu UV-2450 spectrophotometer. Photoluminescence spectra and radiative lifetimes were measured with a steady-state and transient fluorescence spectrometer (Edinburgh FLS-980). Degassed spectra and lifetimes were obtained using a freeze-pump-thaw degassing method: the sample solution was frozen into a solid at 77 K, evacuated under vacuum for 5 minutes, and then backfilled with nitrogen before being allowed to thaw naturally. This procedure was repeated at least three times before measurement. Single crystals of ACRSP and ACRSA were grown by slow diffusion of toluene into CH_2Cl_2 solutions. Single-crystal X-ray diffraction data were collected using a Rigaku XtaLAB Synergy diffractometer. The structures were solved using the SHELXT direct methods in the Olex2 software package and refined by the least-squares method in the ShelXL refinement package. All non-hydrogen atoms were refined anisotropically, and hydrogen atoms were placed in idealized positions using geometric constraints.

5. Synthetic procedures.

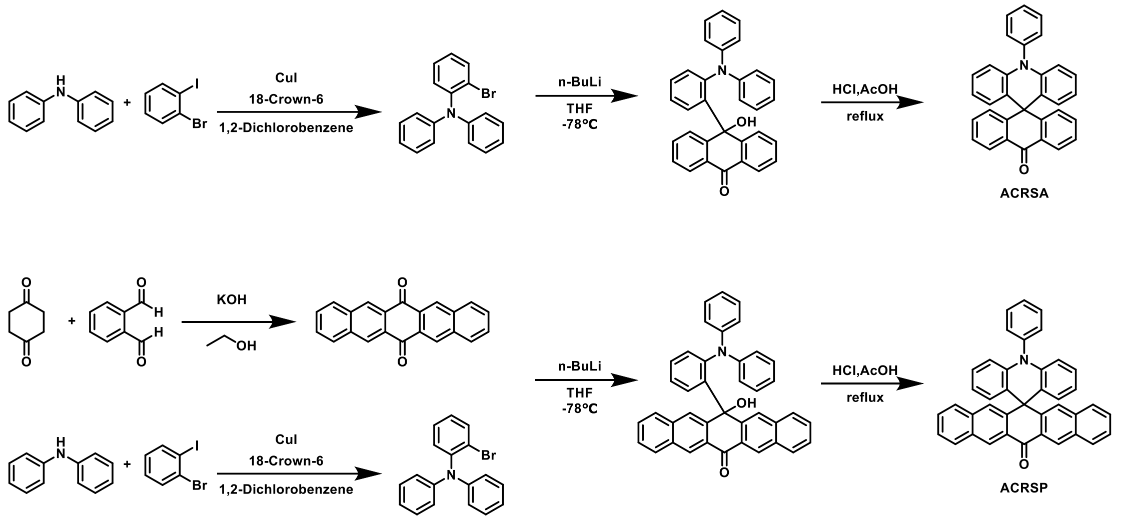

Figure S11. Synthesis procedures, reagents and conditions of **ACRSA** and **ACRSP**.

2-Bromotriphenylamine and 6,13-Pentacenequinone was synthesized according to a published procedure[15c, 43].

Synthesis of Compound 10-phenyl-10H,10'H-spiro[acridine-9,9'-anthracen]-10'-one (ACRSA): ACRSA was synthesized according to a published procedure[16d]. ^{1}H NMR (600 MHz, Chloroform-d) δ 8.40 (dd, J = 8.0, 1.0 Hz, 2H), 7.73 (t, J = 7.6 Hz, 2H), 7.60 (t, J = 7.6 Hz, 1H), 7.51-7.48 (m, 4H), 7.42 (d, J = 8.2 Hz, 2H), 7.37 (t, J = 7.7 Hz, 2H), 6.90-6.87 (m, 2H), 6.57 (t, J = 7.9 Hz, 2H), 6.49 (dd, J = 8.0, 1.4 Hz, 2H), 6.33 (d, J = 8.5 Hz, 2H). ^{13}C NMR (151 MHz, CHLOROFORM-*D*) δ 184.3, 153.5, 141.0, 139.8, 134.3, 132.3, 131.5, 131.4, 131.3, 129.0, 128.8, 127.3, 126.9, 126.8, 126.5, 120.9, 115.1, 48.5. HRMS (ESI) calculated for $C_{40}H_{26}NO$ $[M+H]^+$: 436.1701, found $[M+H]^+$: 436.1696.

Synthesis of Compound 10-phenyl-10H,13'H-spiro[acridine-9,6'-pentacen]-13'-one (ACRSP): In a 250 mL round-bottom flask, n-BuLi (2.5 M in hexane, 5 mL, 12.5 mmol, 1.25 equiv) was added to a solution of 2-bromotriphenylamine (3.87 g, 12 mmol, 1.2 equiv) in anhydrous tetrahydrofuran (30 mL) at -78°C protected by N_2. After stirring at -78°C for 1.5 h, a suspension of 6,13-Pentacenequinone (3.08 g, 10 mmol, 1 equiv) in anhydrous THF (80 ml) was slowly added to the reaction solution and stirred overnight at room temperature. The reaction mixture was poured into water and filtered using a Büchner funnel, the residue was purified by column chromatography (PE/DCM=1:2) on silica gel. The crude product was dissolved in a mixture of glacial acetic acid (55 mL) and HCl (5 mL). After refluxing for 4 h, the mixture was cooled to room temperature and poured into ice water (150 mL). Filtered with a Büchner funnel. The crude product was extracted into DCM. The organic phase was collected, washed

by saturated $NaHCO_3$ aq, and concentrated in vacuo. The product was purified multiple times by column chromatography (PE/DCM=1:1) to afford ACRSP (4.20 g, 7.84 mmol, 78%).^{1}H NMR (600 MHz, Chloroform-*d*, 298K, ppm) δ 9.09 (s, 2H), 8.07 (d, J = 7.8 Hz, 2H), 7.88 (s, 2H), 7.79 (t, J = 7.7 Hz, 2H), 7.70 (d, J = 7.8 Hz, 2H), 7.65-7.61 (m, 3H), 7.51-7.47 (m, 4H), 6.90-6.87 (t, J = 7.6 Hz, 2H), 6.60 (d, J = 7.8 Hz, 2H), 6.53 (t, J = 7.1 Hz, 2H), 6.42 (d, J = 8.4 Hz, 2H). ^{13}C NMR (151 MHz, CHLOROFORM-*D*) δ 184.9, 149.4, 141.2, 139.6, 136.6, 132.1, 131.9, 131.7, 131.5, 131.4, 129.9, 129.5, 128.8, 128.7, 128.6, 128.11, 128.09, 127.1, 126.7, 120.9, 115.0, 48.8. HRMS (ESI) calculated for $C_{40}H_{26}NO$ $[M+H]^+$: 536.2014, found $[M+H]^+$:536.2015.

6. X-ray crystallography.

Table S8. Crystallographic data of **ACRSA** and **ACRSP**.

Compound	**ACRSA**	**ACRSP**
CCDC	2373724	2373723
Empirical formula	$C_{37}H_{21}NO$	$C_{40}H_{25}NO$
Formula Weight	495.55	535.61
Temperature/K	100	100
Crystal System	monoclinic	triclinic
Space Group	$P2_1/n$	P-1
Colour	White	Yellow
a/Å	9.44870(10)	10.6327(2)
b/Å	20.0701(3)	10.6543(2)
c/Å	12.9064(2)	14.4460(2)
α/°	90	76.5520(10)
β/°	94.0260(10)	68.695(2)
γ/°	90	64.880(2)
Volume/Å^3	2441.48(6)	1374.50(5)
Z	4	2
$\rho_{calc.}$/g cm^{-3}	1.348	1.294
μ/mm^{-1}	0.625	0.595
F(000)	1032	560
Crystal size/mm^3	0.15 × 0.12 × 0.12	0.25 × 0.18 × 0.1
Radiation	Cu Kα (λ = 1.54184)	Cu Kα (λ = 1.54184)
2Θ range for data collection/°	8.158 to 151.778	6.596 to 152.418
Index ranges	$-11 \leq h \leq 11$<br>$-17 \leq k \leq 25$<br>$-15 \leq l \leq 16$	$-12 \leq h \leq 13$<br>$-9 \leq k \leq 13$<br>$-17 \leq l \leq 17$
Reflections collected	18071	18131

Independent reflections	4906 [R_{int} = 0.0418, R_{sigma} = 0.0367]	5486 [R_{int} = 0.0318, R_{sigma} = 0.0335]
Data/restraints/parameters	4906/0/307	5486/0/379
Goodness-of-fit on F^2	1.031	1.064
Final R indexes [I>=2σ (I)]	R_1 = 0.0443, wR_2 = 0.1175	R_1 = 0.0426, wR_2 = 0.1221
Final R indexes [all data]	R_1 = 0.0500, wR_2 = 0.1214	R_1 = 0.0470, wR_2 = 0.1264
Largest diff. peak/hole / e $Å^{-3}$	0.24/-0.24	0.25/-0.25

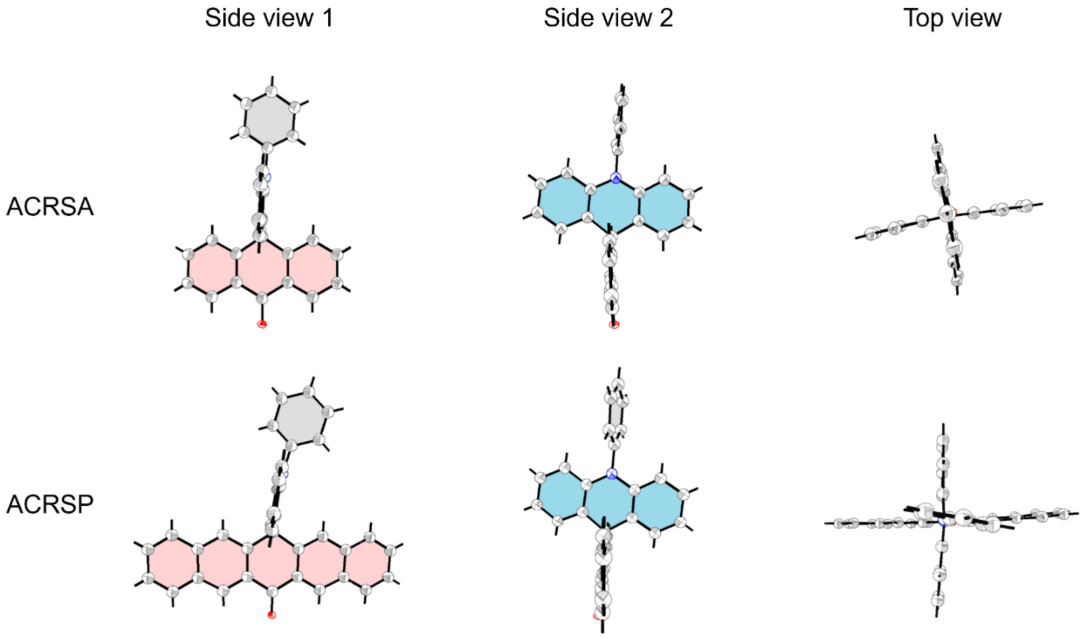

Figure S12. X-ray single crystal diffraction patterns of **ACRSA** and **ACRSP** (from left to right are the acceptor unit view, donor unit view, and top view).

7. ^{1}H, ^{13}C NMR and ESI-MS spectra.

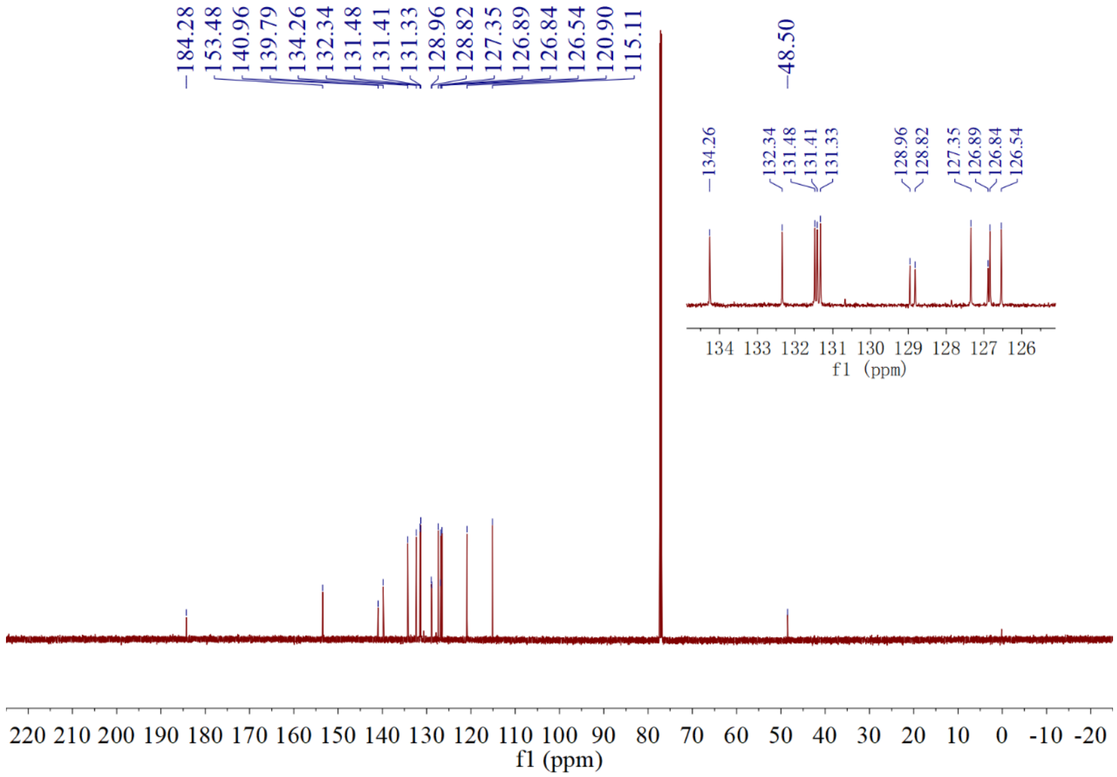

Figure S13. ^{13}C NMR spectrum (151 MHz, $CDCl_3$) of compound **ACRSA**.

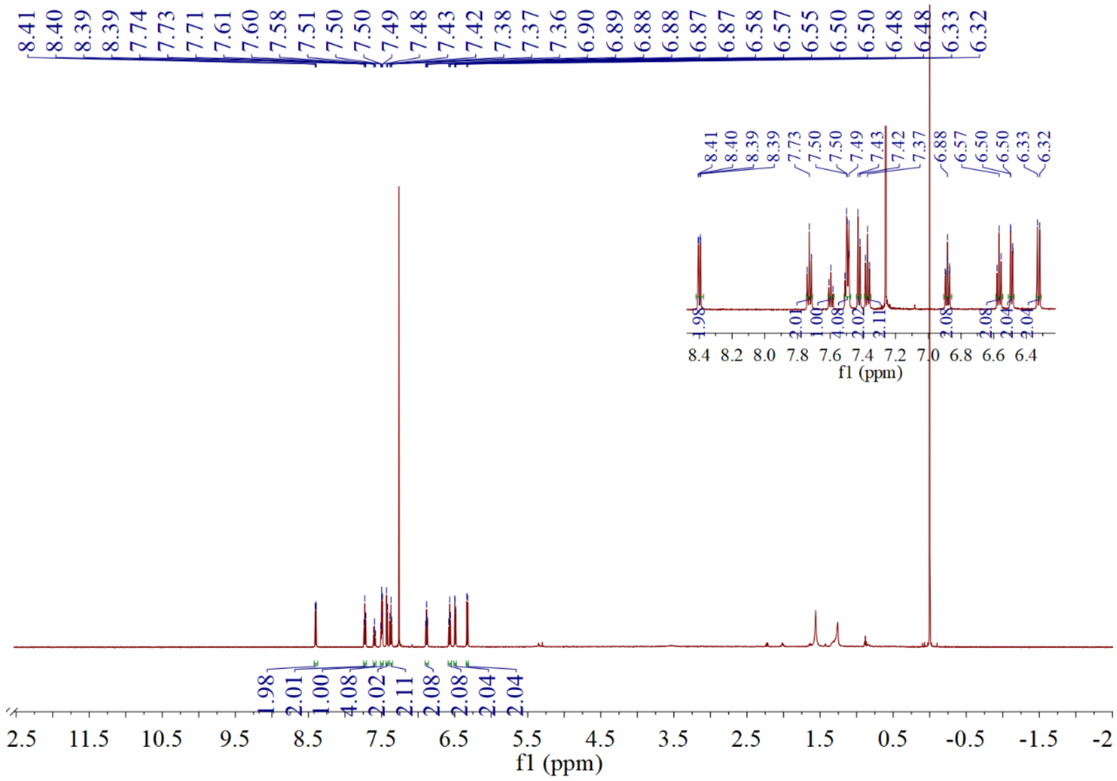

Figure S14. ^{1}H NMR spectrum (600 MHz, $CDCl_3$) of compound **ACRSA**.

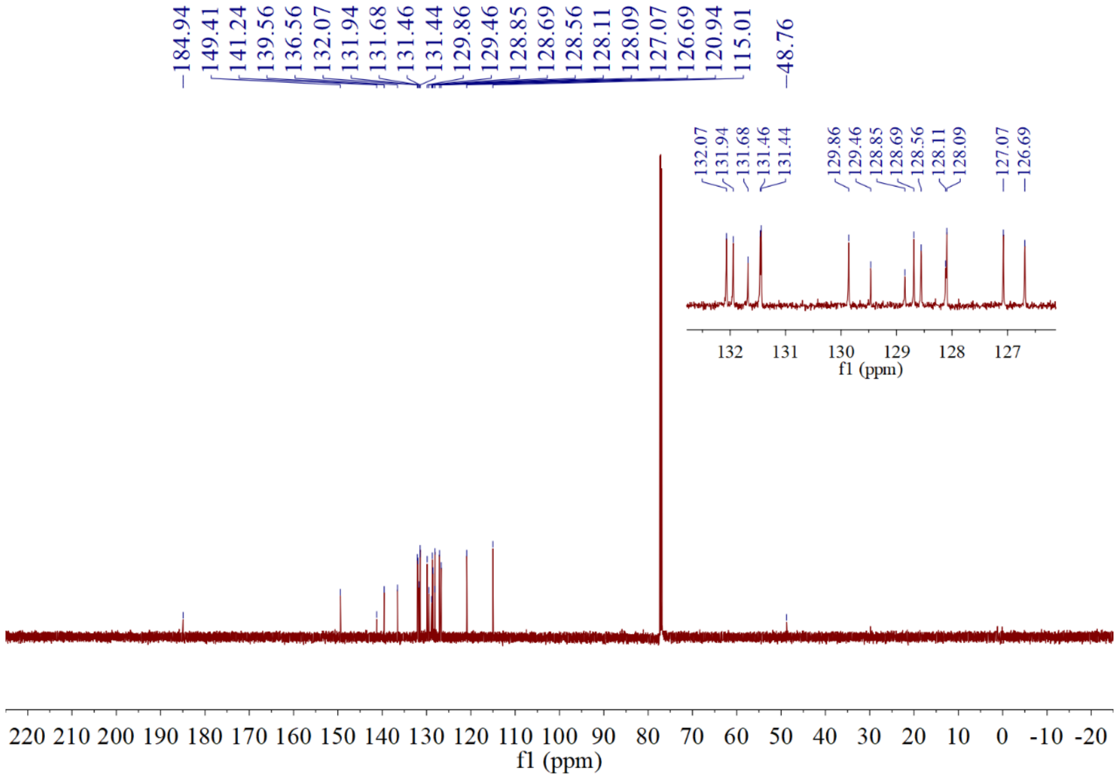

Figure S15. ^{13}C NMR spectrum (151 MHz, $CDCl_3$) of compound **ACRSP**.

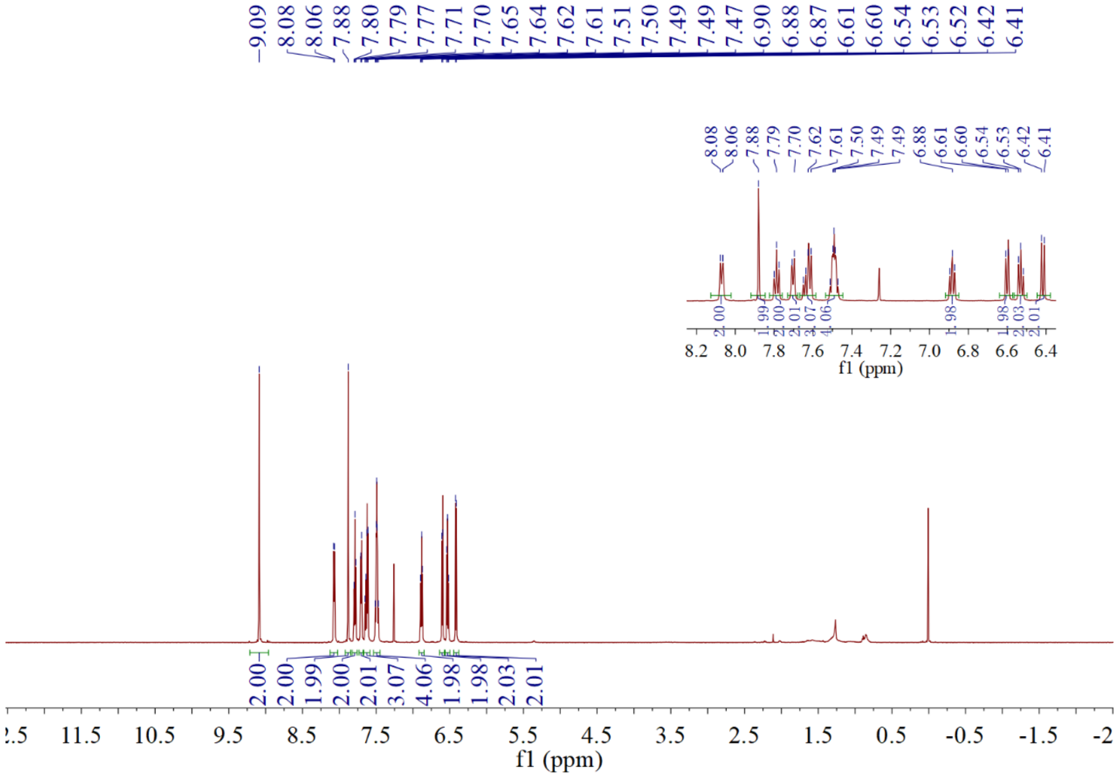

Figure S16. ^{1}H NMR spectrum (600 MHz, $CDCl_3$) of compound **ACRSP**.

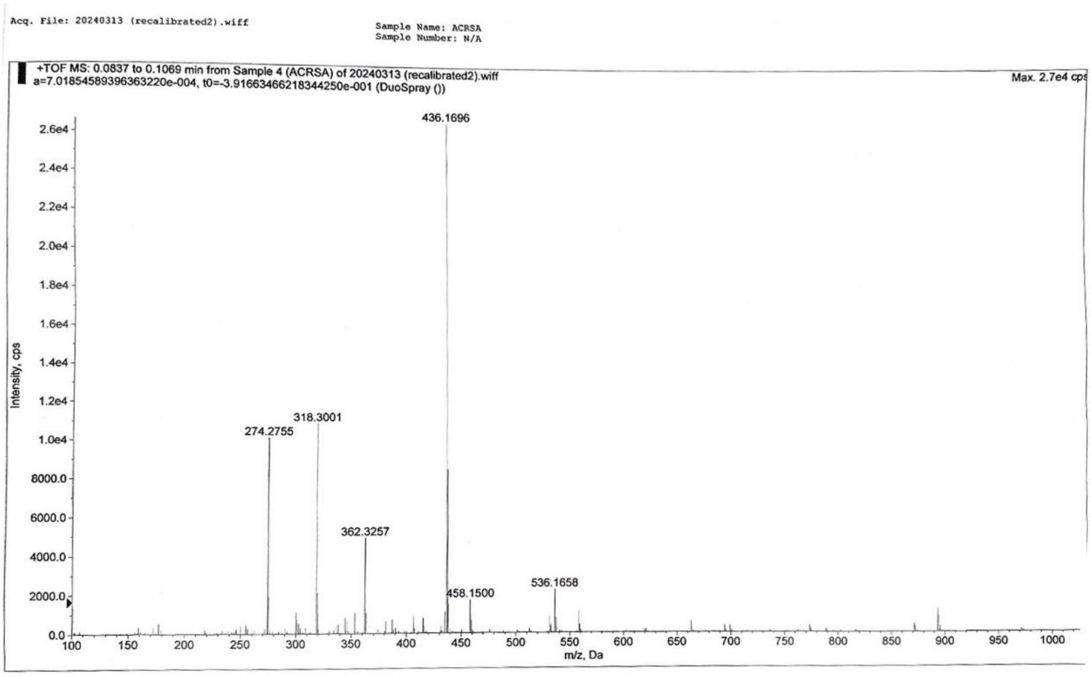

Figure S17. ESI-MS spectrum of **ACRSA**.

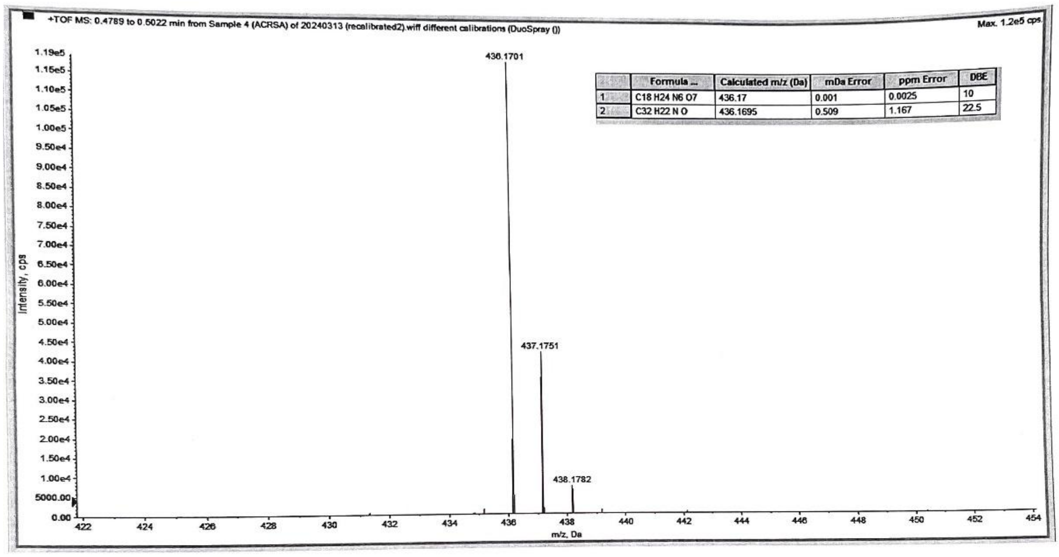

Figure S18. ESI-MS spectrum of **ACRSA** (part).

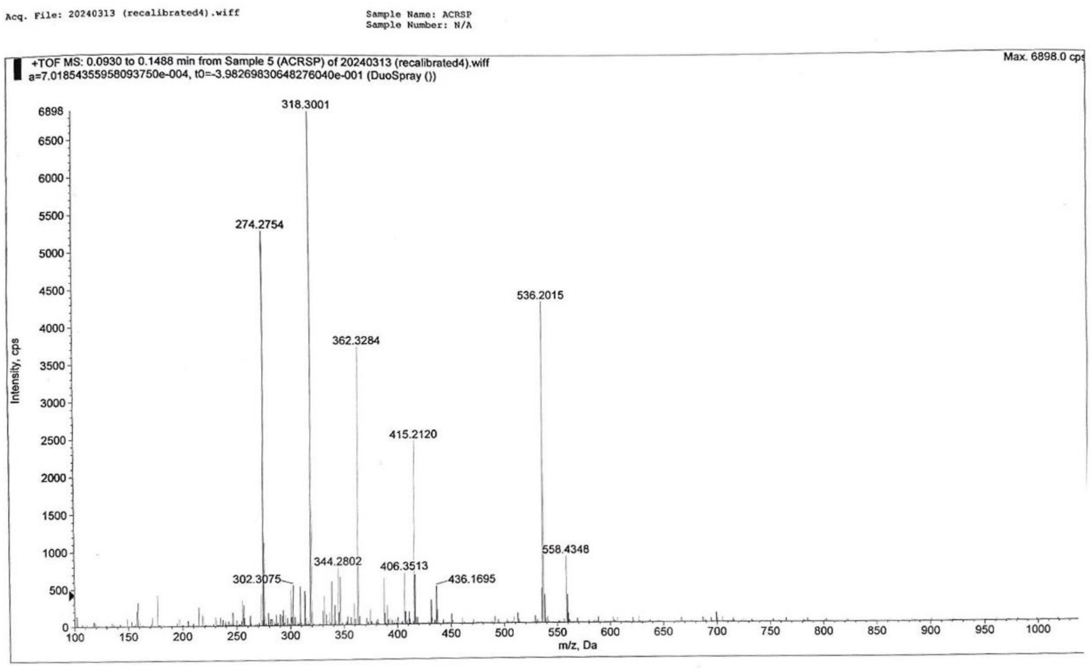

Figure S19. ESI-MS spectrum of **ACRSP**.

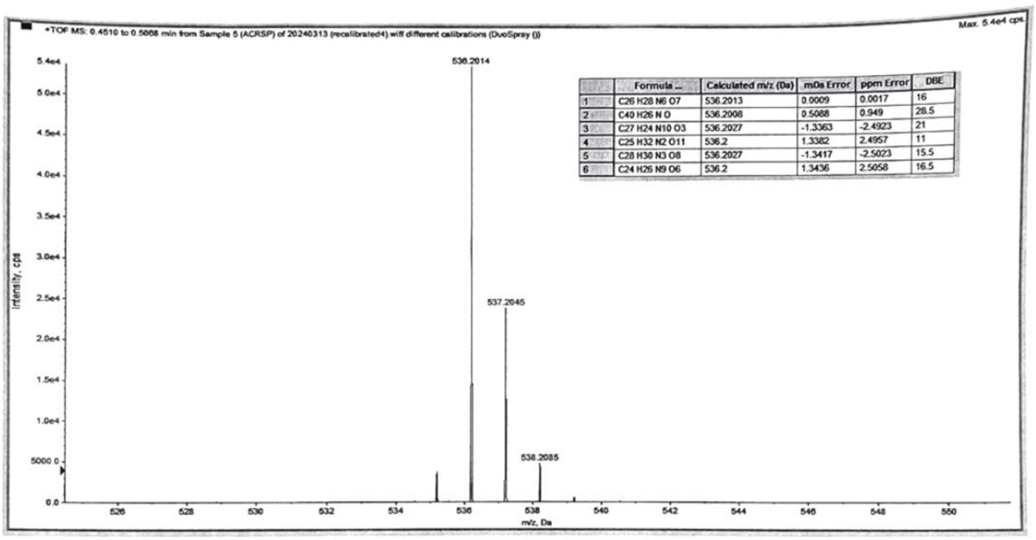

	Formula ...	Calculated m/z (Da)	mDa Error	ppm Error	DBE
1	C26 H28 N6 O7	536.2013	0.0009	0.0017	16
2	C40 H26 N O	536.2008	0.5088	0.949	28.5
3	C27 H24 N10 O3	536.2027	-1.3363	-2.4923	21
4	C25 H32 N2 O11	536.2	1.3382	2.4957	11
5	C28 H30 N3 O8	536.2027	-1.3417	-2.5023	15.5
6	C24 H26 N9 O6	536.2	1.3436	2.5058	16.5

Figure S20. ESI-MS spectrum of **ACRSP** (part).

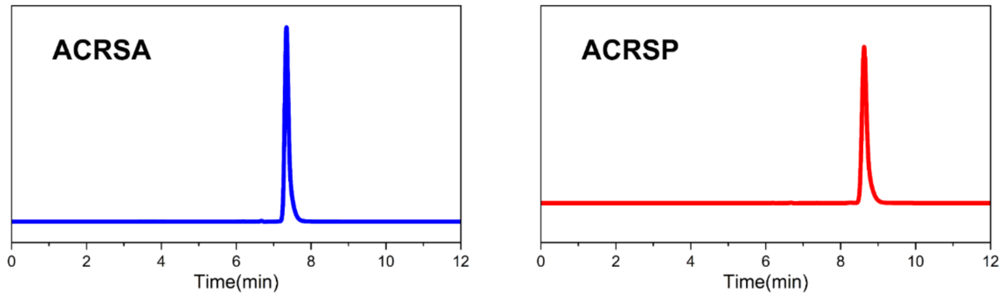

Figure S21. HPLC spectrum of **ACRSA** and **ACRSP**.

8. Photophysical properties of ACRSP.

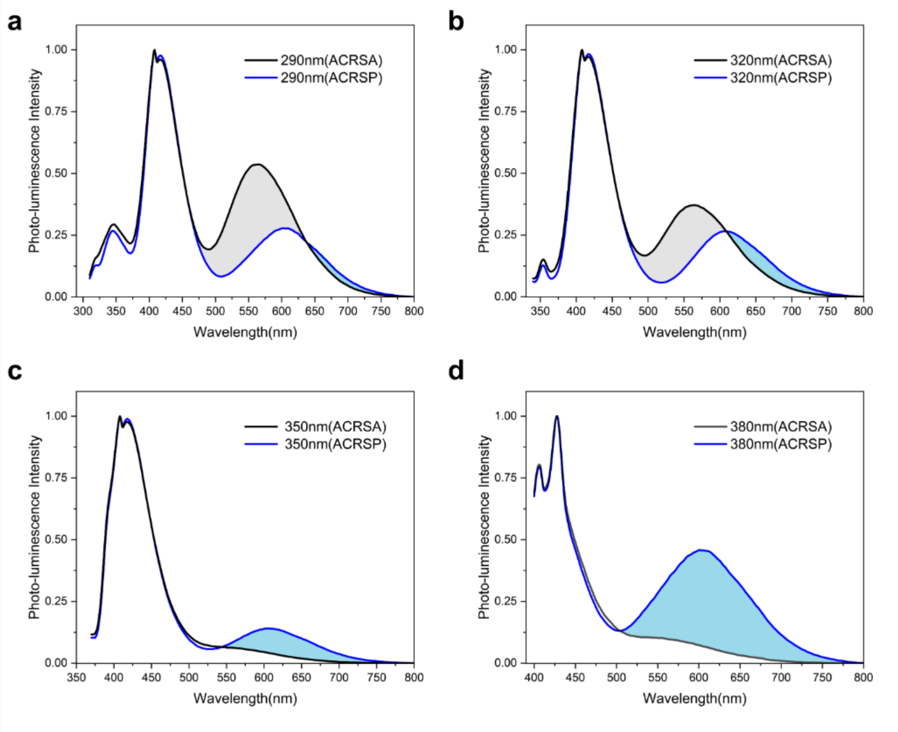

Figure S22. The excitation wavelength dependent emission spectra of **ACRSP** and **ACRSA** excited at 290nm(**a**), 320nm(**b**), 350nm(**c**) and 380nm(**d**) in DMF (10 μM).

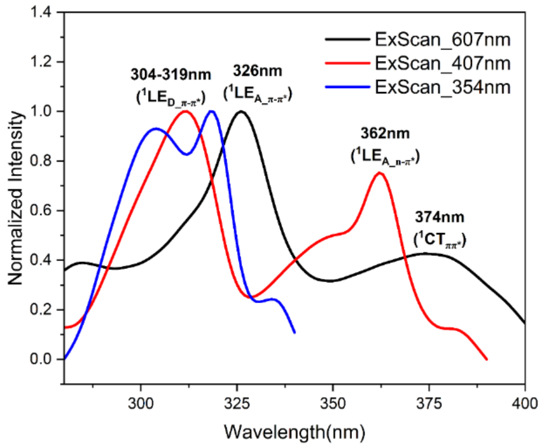

Figure S23. Excitation spectra of $^{1}CT_{\pi\pi*}$(607 nm),$^{1}LE_{A\_n\pi*}$(407 nm), and $^{1}LE_{D\_\pi\pi*}$(354 nm) of **ACRSP** in DMF (10 μM).

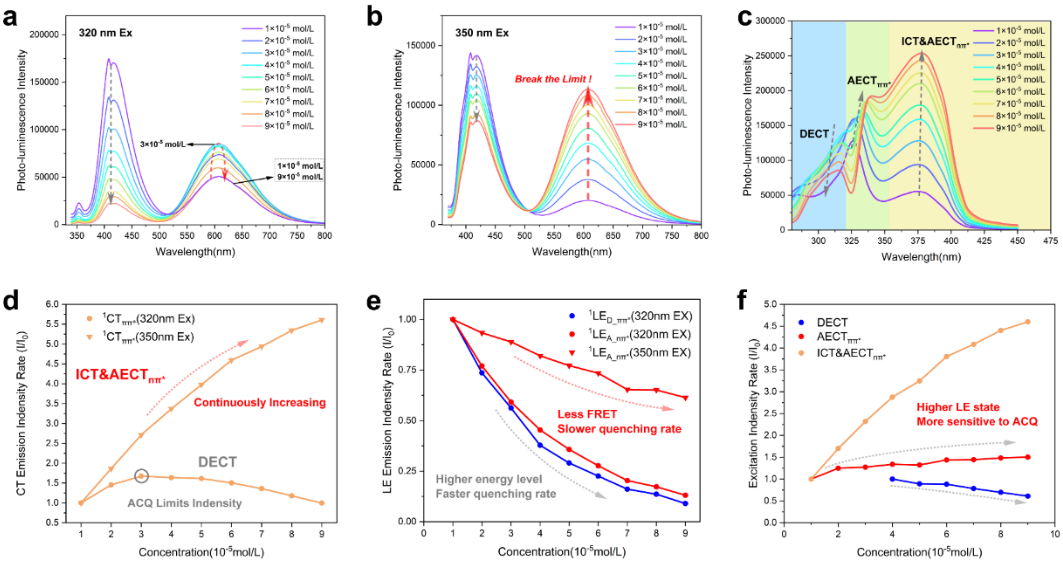

Figure S24. **a-b**, Emission spectra of ACRSP in DMF at 320 nm (**a**) and 350 nm (**b**) excitation wavelengths across different concentrations (10 μM to 90 μM). **c**, Excitation spectra of ACRSP in DMF at various concentrations. **d**. The luminescence intensity curves of $^1CT_{\pi\pi^*}$ emission peaks of **ACRSP** at different concentrations under excitation wavelengths of 320nm and 350nm. **e**. The luminescence intensity curves of $^1LE_{D\_\pi\pi^*}$ and $^1LE_{A\_n\pi^*}$ emission peaks of **ACRSP** at different concentrations under excitation wavelengths of 320nm and 350nm. **f**. The excitation intensity curves of different modes of **ACRSP** at different concentrations.

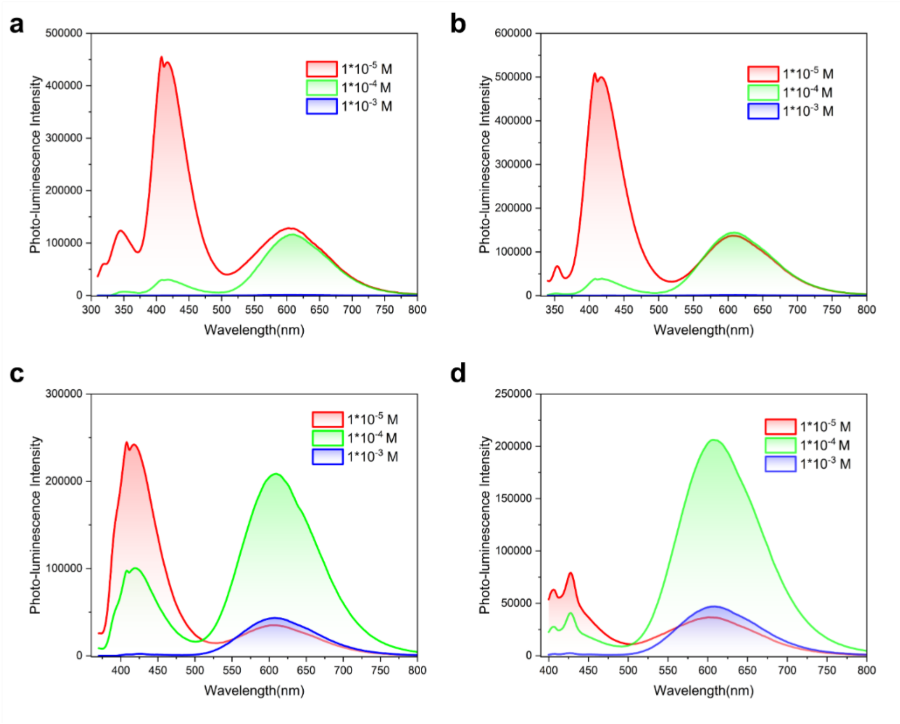

Figure S25. Concentration dependent emission spectra of **ACRSP** excited at 290nm(**a**), 320nm(**b**), 350nm(**c**) and 380nm(**d**) in DMF (from 10 μM to 1 mM).

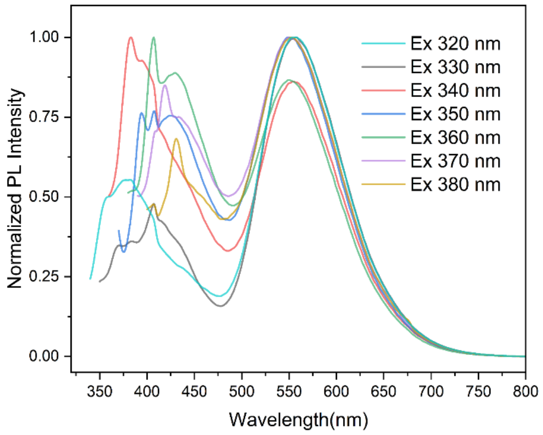

Figure S26. Emission spectra of **ACRSP** at multiple excitation wavelengths in o-DCB.

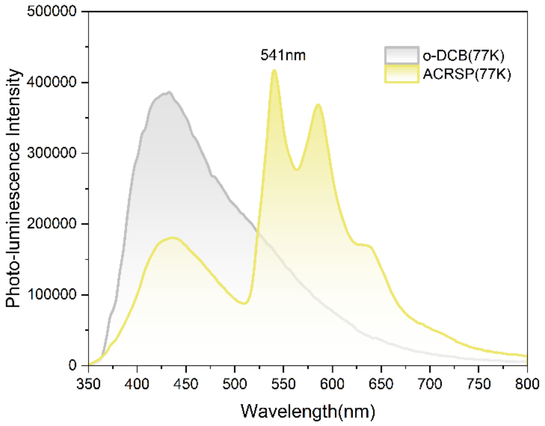

Figure S27. 77K phosphorescence spectra of ACRSP in o-DCB solution at excitation wavelengths of 330nm (10ms delay).

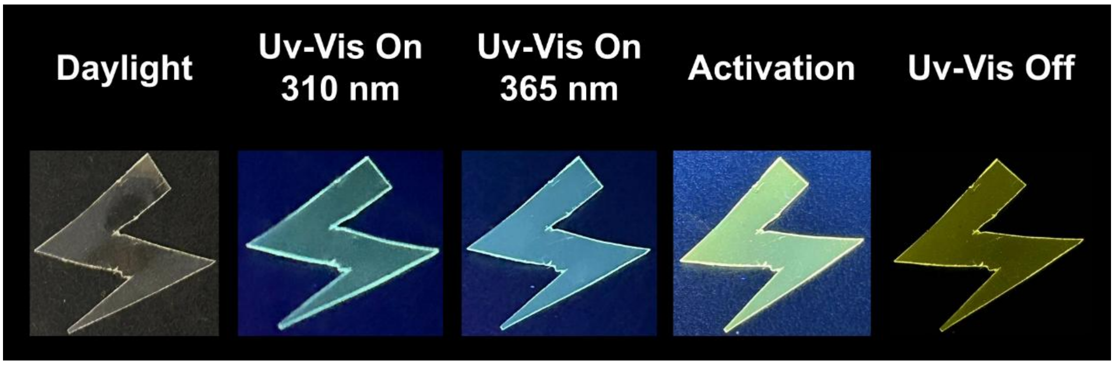

Figure S28. Ex-De RTP properties of **ACRSP-0.1%-PMMA**.

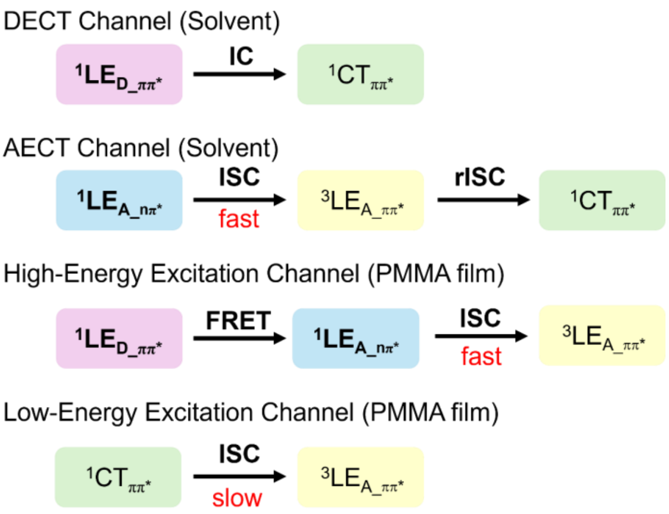

Figure S29. Differences in relaxation pathways between solutions and PMMA films of **ACRSP.**

9. The critical points for the minimum energy profile (MEP) of NEVPT2 // CASSCF calculations.

Optimized S_0 geometry of compound Phenyl Acridine

Center Number	Atomic Number	Coordinates (Angstroms) X	Y	Z
1	C	-0.4974312	-0.2399101	3.7157068
2	C	-0.0278786	0.3005123	2.5236984
3	C	-0.4091353	-0.1954107	1.2645261
4	C	-1.3034782	-1.2837532	1.2232076
5	C	-1.7725531	-1.8230506	2.4338453
6	C	-1.3837174	-1.3192056	3.6509325
7	C	-1.2725949	-1.3114709	-1.2204786
8	C	-0.3775924	-0.2240202	-1.2636587
9	C	0.0351234	0.2436143	-2.5238243
10	H	0.7210742	1.0770297	-2.57605
11	C	-0.404325	-0.3237875	-3.7147303
12	C	-1.2918842	-1.4018953	-3.6479873
13	C	-1.7111212	-1.8783944	-2.4299851
14	H	-0.1820657	0.1681384	4.6663361
15	H	0.6570212	1.1349752	2.5739077
16	H	-2.4576786	-2.655272	2.4154666
17	H	-1.7704357	-1.7662625	4.5578472
18	H	-0.0652146	0.0627741	-4.6661834
19	H	-1.6556271	-1.869326	-4.5541566

20	H	-2.3962404	-2.7104696	-2.4102401
21	N	-1.7250487	-1.8269751	0.0019115
22	C	0.1575292	0.462473	-0.0002481
23	C	-0.257032	1.9502875	-0.0225435
24	H	0.1360429	2.4581201	-0.9035452
25	H	-1.3436509	2.0478151	-0.034722
26	H	0.1187563	2.4801545	0.8531289
27	C	1.6978371	0.3484378	0.0199845
28	H	2.1207581	0.8398901	0.8965319
29	H	2.0070289	-0.6977315	0.0382645
30	H	2.1414472	0.8148593	-0.8598849
31	C	-2.6282184	-2.9310673	0.0025955
32	C	-2.1404866	-4.2430398	0.0237172
33	C	-4.0080933	-2.7149268	-0.0185843
34	C	-3.0264978	-5.3197329	0.022924
35	H	-1.0715314	-4.4087928	0.0406093
36	C	-4.8880034	-3.7976631	-0.0192124
37	H	-4.383318	-1.7003786	-0.0344617
38	C	-4.4025557	-5.0914413	0.0010015
39	H	-2.6443341	-6.3321867	0.0395221
40	H	-5.9558401	-3.6197855	-0.0356268
41	H	-5.0893554	-5.9282886	0.0001423

Optimized ^{1}LE$_{D_\pi\pi*(1)}$ geometry of compound Phenyl Acridine

Center Number	Atomic Number	Coordinates (Angstroms) X	Y	Z
1	C	-0.4571383	-0.2985591	3.7004963
2	C	0.0093304	0.2581948	2.5190103
3	C	-0.3672619	-0.2102465	1.25512
4	C	-1.2854073	-1.3240292	1.2098401
5	C	-1.7617001	-1.8796768	2.430447
6	C	-1.3605067	-1.3877247	3.6396398
7	C	-1.2505313	-1.3545259	-1.206957
8	C	-0.3341103	-0.2394631	-1.2539009
9	C	0.0726764	0.2023432	-2.5180996
10	H	0.7551353	1.0375187	-2.5847749
11	C	-0.3620835	-0.3821209	-3.6982095
12	C	-1.2646419	-1.4719765	-3.6358077
13	C	-1.695201	-1.9379322	-2.4263736
14	H	-0.1356908	0.0976646	4.6532857
15	H	0.6932929	1.0922256	2.5843742
16	H	-2.4498857	-2.7079797	2.4076932
17	H	-1.7393625	-1.8351253	4.5484129

18	H	-0.0174882	-0.006341	-4.6511909
19	H	-1.620016	-1.9400991	-4.5436253
20	H	-2.3853525	-2.7645751	-2.4026249
21	N	-1.6837187	-1.8247454	0.0016872
22	C	0.1471129	0.4561507	-0.0007289
23	C	-0.3689577	1.9238301	-0.023751
24	H	-0.0070431	2.451218	-0.9061732
25	H	-1.4587889	1.9471744	-0.0370518
26	H	-0.0281902	2.4711457	0.8549641
27	C	1.6983523	0.4584049	0.0189693
28	H	2.079076	0.9810451	0.8953054
29	H	2.0841949	-0.56097	0.0349622
30	H	2.1010419	0.9617203	-0.8588658
31	C	-2.6231323	-2.9118234	0.0020166
32	C	-2.146104	-4.2234647	0.0199316
33	C	-3.9922093	-2.6371911	-0.0156763
34	C	-3.0660705	-5.2758667	0.0199559
35	H	-1.0815816	-4.4084344	0.0326462
36	C	-4.8974257	-3.7035041	-0.0157205
37	H	-4.3341126	-1.6120816	-0.0282588
38	C	-4.4226287	-5.0190513	0.0022377
39	H	-2.7080189	-6.2967137	0.0342964
40	H	-5.960619	-3.5077679	-0.0303718
41	H	-5.1255547	-5.8418041	0.0025543

Optimized S_0 geometry of compound pentacene ketone

Center Number	Atomic Number	Coordinates (Angstroms) X	Y	Z
1	C	-0.954228	-3.4723599	-2.58968
2	C	0.2438429	-3.6088929	-1.8547876
3	C	0.6620179	-2.6156228	-0.9779068
4	C	-0.1062401	-1.4339331	-0.8070833
5	C	-1.2682501	-1.2949082	-1.5157312
6	C	-1.7270951	-2.2915997	-2.4163066
7	C	1.9329559	-2.849053	-0.2234663
8	C	0.3428572	-0.3250938	0.1169657
9	C	1.5553908	-0.622798	0.9689666
10	C	2.3157355	-1.8104531	0.7898443
11	C	3.438336	-2.0498057	1.5643864
12	H	3.993747	-2.9620233	1.4025265
13	C	3.8524548	-1.126642	2.553094
14	C	3.0919384	0.0573576	2.7349526
15	C	1.9506639	0.2734302	1.923322

16	H	0.8530393	-4.4943498	-1.9608357
17	H	-1.8633971	-0.3976833	-1.3940037
18	H	1.3792078	1.1825474	2.069588
19	C	3.5040114	0.9967497	3.734672
20	C	5.0064861	-1.3464319	3.371883
21	H	5.5836336	-2.2511792	3.2310019
22	C	5.3712513	-0.4269737	4.3187607
23	H	6.2451344	-0.5925549	4.9348115
24	C	4.5988472	0.7647714	4.4954667
25	C	-1.414777	-4.4782856	-3.498784
26	H	-0.8249565	-5.3765026	-3.6280387
27	C	-2.9390781	-2.1476963	-3.1547894
28	C	-2.5860508	-4.3057258	-4.1914484
29	H	-2.9310842	-5.065274	-4.8803499
30	C	-3.3541372	-3.1165172	-4.0106134
31	H	-4.2755236	-2.991502	-4.5648058
32	H	-3.527756	-1.2489144	-3.0208248
33	H	2.9217994	1.8990119	3.8734374
34	H	4.9001113	1.484058	5.2462984
35	O	2.5912991	-3.8133158	-0.4006075
36	H	-0.4884261	-0.0518593	0.7688823
37	H	0.5555287	0.560341	-0.4878319

Optimized $^{1}LE_{A\_n\pi^*}$ geometry of compound pentacene ketone

Center Number	Atomic Number	Coordinates (Angstroms) X	Y	Z
1	C	-0.9599043	-3.4532417	-2.5835484
2	C	0.231525	-3.5908483	-1.8468199
3	C	0.6538038	-2.6090667	-0.9772648
4	C	-0.115161	-1.4273376	-0.814197
5	C	-1.2749296	-1.2929653	-1.5301785
6	C	-1.7264476	-2.2876954	-2.4237474
7	C	1.8778359	-2.7529298	-0.1956291
8	C	0.3382865	-0.3147649	0.1114602
9	C	1.5489331	-0.609407	0.9781497
10	C	2.3218096	-1.8334823	0.7739547
11	C	3.4670958	-2.0565733	1.5596767
12	H	4.0468808	-2.9578434	1.4179919
13	C	3.8740833	-1.1271347	2.5513403
14	C	3.1055107	0.0496133	2.7471667
15	C	1.9431716	0.2615587	1.9246004
16	H	0.8167696	-4.4918727	-1.9694723
17	H	-1.8696678	-0.3947267	-1.4125463

18	H	1.3732244	1.1701889	2.0819408
19	C	3.5018652	0.9785877	3.7418166
20	C	5.0266496	-1.3379822	3.3659949
21	H	5.6163114	-2.2342238	3.2214335
22	C	5.383686	-0.4179317	4.3231858
23	H	6.259028	-0.5833719	4.9373813
24	C	4.6056439	0.7573389	4.5086713
25	C	-1.4171043	-4.4764576	-3.4925683
26	H	-0.8215236	-5.37229	-3.6107103
27	C	-2.9497855	-2.143545	-3.1722394
28	C	-2.5812636	-4.317376	-4.1899806
29	H	-2.9196443	-5.0886698	-4.8694038
30	C	-3.3633914	-3.1272024	-4.0267944
31	H	-4.2828881	-3.0125447	-4.5857327
32	H	-3.5357475	-1.2424635	-3.0452474
33	H	2.9113199	1.8748832	3.8856143
34	H	4.8938717	1.4745014	5.2661801
35	O	2.5890518	-3.8926578	-0.3882277
36	H	-0.4964444	-0.036032	0.7565629
37	H	0.5568352	0.5662817	-0.497849

Optimized S_0 geometry of compound **ACRSP**

Center Number	Atomic Number	Coordinates (Angstroms)		
		X	Y	Z
1	C	-4.6309871	-2.8352671	-2.8170497
2	C	-3.5182751	-2.9820949	-1.9731371
3	C	-3.1183846	-1.967694	-1.129497
4	C	-3.822206	-0.7322375	-1.0852212
5	C	-4.9014945	-0.5858882	-1.9016234
6	C	-5.3359708	-1.6126053	-2.7804862
7	C	-1.925045	-2.2220662	-0.2723266
8	C	-3.4080878	0.4350497	-0.1539941
9	C	-2.1660388	0.1068509	0.7081221
10	C	-1.4843359	-1.1395544	0.6403805
11	C	-0.3731428	-1.3855842	1.4340815
12	H	0.1162395	-2.3446671	1.3531345
13	C	0.1137252	-0.4200612	2.3267597
14	C	-0.5586636	0.8222961	2.3993271
15	C	-1.6962287	1.0454144	1.5724624
16	H	-2.9554551	-3.9037982	-1.9750911
17	H	-5.4604896	0.3389988	-1.8947216
18	H	-2.1930561	2.0024006	1.6466469
19	C	-0.0760175	1.8081619	3.2992062

20	C	1.2579391	-0.6480873	3.1555166
21	H	1.7652502	-1.6020031	3.0921589
22	C	1.6942823	0.3141155	4.0053761
23	H	2.5586853	0.1408742	4.6325734
24	C	1.0159081	1.562798	4.076511
25	C	-5.0643796	-3.8733628	-3.7011846
26	H	-4.5150433	-4.8056871	-3.7194048
27	C	-6.4662964	-1.4516619	-3.6310408
28	C	-6.1440506	-3.6897998	-4.4991444
29	H	-6.4733425	-4.4744305	-5.1671358
30	C	-6.8551275	-2.4564003	-4.4604473
31	H	-7.71479	-2.321708	-5.1041826
32	H	-7.0121113	-0.5177073	-3.6087742
33	H	-0.5868994	2.7601893	3.3586918
34	H	1.375422	2.3228082	4.7580401
35	O	-1.340694	-3.2761453	-0.3235295
36	C	-5.9869171	-0.0140635	2.6245531
37	C	-4.9129006	-0.1865687	1.7771768
38	C	-4.5878656	0.7377046	0.7836231
39	C	-5.3734856	1.8825652	0.6496069
40	C	-6.4703539	2.0580493	1.5125368
41	C	-6.7703043	1.1247793	2.4807686
42	C	-3.9218946	2.7665926	-1.1047786
43	C	-3.0842163	1.6534195	-1.0334584
44	C	-1.9337696	1.6278253	-1.8226992
45	H	-1.2813029	0.7678795	-1.761058
46	C	-1.6000798	2.6576567	-2.6767543
47	C	-2.4429318	3.760241	-2.7491071
48	C	-3.5829068	3.8165242	-1.9772813
49	H	-6.2107675	-0.7516612	3.3828948
50	H	-4.2988397	-1.0699548	1.8848659
51	H	-7.0909778	2.9337778	1.4208436
52	H	-7.6224733	1.2894368	3.1273699
53	H	-0.7010219	2.6039629	-3.2751298
54	H	-2.2119964	4.5845533	-3.4113548
55	H	-4.2202553	4.6820432	-2.0490177
56	C	-5.9362545	3.9884137	-0.455333
57	C	-5.7026823	5.1247638	0.3106822
58	C	-7.0011768	3.9693788	-1.3460407
59	C	-6.5282587	6.2326563	0.1856854
60	H	-4.8717344	5.134662	1.003023
61	C	-7.8281341	5.0778709	-1.4712549
62	H	-7.1781196	3.0827033	-1.9392458

63	C	-7.5930706	6.211064	-0.705788
64	H	-6.3403469	7.1132671	0.7857574
65	H	-8.6556284	5.0549878	-2.1680651
66	H	-8.2370132	7.0752197	-0.8030281
67	N	-5.0890825	2.8448749	-0.3306314

Optimized [1]CT$_{\pi\pi*}$ geometry of compound **ACRSP**

Center Number	Atomic Number	Coordinates (Angstroms) X	Y	Z
1	C	-4.5624407	-2.9752639	-2.8376514
2	C	-3.4302899	-3.0612208	-2.0039177
3	C	-3.0496262	-2.0196115	-1.15039
4	C	-3.8436897	-0.8408605	-1.1385637
5	C	-4.9530607	-0.7319774	-1.9417439
6	C	-5.3561547	-1.7827295	-2.8156842
7	C	-1.864021	-2.1912187	-0.3132718
8	C	-3.4759874	0.3510342	-0.2409732
9	C	-2.2496406	0.1209384	0.6559634
10	C	-1.5079551	-1.0901152	0.5833325
11	C	-0.3917387	-1.229571	1.4120031
12	H	0.1600954	-2.1683065	1.3382974
13	C	0.0131512	-0.2187333	2.3052808
14	C	-0.7422843	0.9967731	2.3711489
15	C	-1.8757667	1.1218209	1.5187991
16	H	-2.8053743	-3.9559197	-1.9957257
17	H	-5.5573682	0.1800223	-1.9265716
18	H	-2.4516009	2.0510053	1.5675904
19	C	-0.3457553	2.0151129	3.2627308
20	C	1.1469946	-0.3489721	3.1538221
21	H	1.7274034	-1.2733561	3.1090881
22	C	1.5098022	0.6636074	4.0146832
23	H	2.3846771	0.5418506	4.6577573
24	C	0.7619963	1.8584818	4.0750352
25	C	-4.9595297	-4.0268696	-3.7083926
26	H	-4.3593349	-4.9393755	-3.7303687
27	C	-6.4915278	-1.6870914	-3.6456147
28	C	-6.0748546	-3.9041226	-4.5083029
29	H	-6.3611726	-4.7253511	-5.1697074
30	C	-6.8521751	-2.7276528	-4.4823418
31	H	-7.7339585	-2.6414098	-5.1203681
32	H	-7.0883598	-0.7710548	-3.6184909
33	H	-0.9315639	2.9377001	3.3029133
34	H	1.0587877	2.6540671	4.7613402

35	O	-1.1887209	-3.232844	-0.3529493
36	C	-6.1520232	-0.1517608	2.4008277
37	C	-5.068989	-0.3576642	1.5559063
38	C	-4.6573731	0.6239221	0.6583025
39	C	-5.369074	1.8442993	0.6244091
40	C	-6.4748977	2.0544375	1.4871212
41	C	-6.8567859	1.0630272	2.3630713
42	C	-3.9150946	2.7131881	-1.1288124
43	C	-3.1615322	1.5185461	-1.1470423
44	C	-2.0949159	1.4234699	-2.0356363
45	H	-1.5148643	0.4997408	-2.0481042
46	C	-1.7719933	2.4723007	-2.8870004
47	C	-2.5233288	3.6575095	-2.8653167
48	C	-3.585803	3.7831272	-1.9970647
49	H	-6.4544245	-0.9372225	3.0948753
50	H	-4.5172304	-1.2982257	1.578731
51	H	-7.0174266	2.9972078	1.4541513
52	H	-7.7075589	1.2259843	3.0259756
53	H	-0.9306655	2.3713483	-3.574189
54	H	-2.2707417	4.481933	-3.5333771
55	H	-4.1719155	4.6998521	-1.9759639
56	C	-5.7320545	4.0669009	-0.2668405
57	C	-5.344224	5.1091014	0.5713481
58	C	-6.8292174	4.1878589	-1.115807
59	C	-6.073142	6.2958188	0.5565173
60	H	-4.4794318	4.9842755	1.2252866
61	C	-7.5510353	5.3789553	-1.1225239
62	H	-7.1081941	3.3534226	-1.7615231
63	C	-7.1740331	6.4306169	-0.2883745
64	H	-5.7774989	7.1186979	1.2095585
65	H	-8.4125054	5.4839329	-1.7842191
66	H	-7.7424148	7.3623822	-0.2968815
67	N	-4.9845585	2.8403158	-0.2558132

Optimized $^{1}LE_{A\_n\pi*}$ geometry of compound **ACRSP**

Center Number	Atomic Number	Coordinates (Angstroms) X	Y	Z
1	C	-4.5973387	-2.7710173	-2.8447816
2	C	-3.4192692	-2.8804171	-2.0934638
3	C	-3.0458836	-1.8714589	-1.1985075
4	C	-3.8474216	-0.6919791	-1.02765
5	C	-4.9855945	-0.5954943	-1.7479099
6	C	-5.4054625	-1.6070842	-2.6696181

7	C	-1.8489522	-2.0323033	-0.4198018
8	C	-3.4169161	0.456495	-0.1000735
9	C	-2.1860718	0.116242	0.7502469
10	C	-1.4306891	-1.089838	0.5464706
11	C	-0.2638825	-1.3559294	1.3126807
12	H	0.2983652	-2.2727635	1.1495142
13	C	0.1708685	-0.4511673	2.2852348
14	C	-0.585297	0.7466588	2.4903932
15	C	-1.7615303	0.9806763	1.6890999
16	H	-2.7956143	-3.7658203	-2.2051707
17	H	-5.6134883	0.2878358	-1.6345997
18	H	-2.3234719	1.9002672	1.8534446
19	C	-0.1542682	1.6570038	3.4662357
20	C	1.3364128	-0.6809626	3.078299
21	H	1.9087649	-1.5950736	2.9185928
22	C	1.728484	0.2281266	4.0211256
23	H	2.6193771	0.0448305	4.6217172
24	C	0.9783928	1.4082981	4.2189186
25	C	-5.00955	-3.7807496	-3.7644838
26	H	-4.3858763	-4.6660623	-3.8907247
27	C	-6.5955662	-1.4938095	-3.4181102
28	C	-6.1657137	-3.6399146	-4.4737765
29	H	-6.4764385	-4.4134254	-5.1753346
30	C	-6.9661357	-2.4833796	-4.2971379
31	H	-7.888669	-2.3807617	-4.8681342
32	H	-7.2163704	-0.6076584	-3.2868227
33	H	-0.7309786	2.5691684	3.6217227
34	H	1.2981267	2.1274169	4.9727499
35	O	-1.1387874	-3.1270848	-0.6094202
36	C	-6.0044449	0.0697744	2.6699805
37	C	-4.9136024	-0.1229593	1.835172
38	C	-4.5865248	0.7804488	0.8238948
39	C	-5.3811548	1.9213174	0.6491717
40	C	-6.4896657	2.1174849	1.4947164
41	C	-6.7943013	1.2022742	2.4888263
42	C	-3.9169958	2.7588347	-1.1133762
43	C	-3.0691668	1.6473865	-0.993465
44	C	-1.9033527	1.6034178	-1.7586264
45	H	-1.2478758	0.7390472	-1.6563782
46	C	-1.5631663	2.6167369	-2.6421548
47	C	-2.413699	3.7126926	-2.7650634
48	C	-3.5729516	3.7868648	-2.0110524
49	H	-6.2377071	-0.6545743	3.4488013

50	H	-4.2878878	-1.0064232	1.9596887
51	H	-7.1133652	2.9976643	1.3657943
52	H	-7.6585816	1.3796291	3.12865
53	H	-0.6480126	2.5518465	-3.2286353
54	H	-2.1729425	4.5233707	-3.4524808
55	H	-4.2244393	4.6503936	-2.1120681
56	C	-5.9377461	3.9801215	-0.5247508
57	C	-5.721984	5.1399843	0.2192198
58	C	-6.9885675	3.9255298	-1.4384396
59	C	-6.5568394	6.2402152	0.0493422
60	H	-4.8956995	5.1671237	0.9284267
61	C	-7.8225644	5.0273388	-1.6075407
62	H	-7.1413855	3.0129787	-2.0130263
63	C	-7.6076807	6.1849163	-0.8638305
64	H	-6.3862915	7.1450597	0.6320157
65	H	-8.6431816	4.9815982	-2.3229433
66	H	-8.260703	7.0471098	-0.9962214
67	N	-5.0870659	2.8500209	-0.3542205

Optimized $^{3}LE_{A\_\pi\pi*}$ geometry of compound **ACRSP**

Center Number	Atomic Number	Coordinates (Angstroms)		
		X	Y	Z
1	C	-4.6720234	-2.8167805	-2.8423746
2	C	-3.5487818	-2.9835409	-1.9989809
3	C	-3.1299661	-1.9699002	-1.1395502
4	C	-3.8276543	-0.7410664	-1.0934599
5	C	-4.9292973	-0.5621825	-1.9123151
6	C	-5.3789882	-1.5753242	-2.7968502
7	C	-1.9453082	-2.2240574	-0.2891088
8	C	-3.4024226	0.3968428	-0.1676095
9	C	-2.1831256	0.0595359	0.6710848
10	C	-1.5181082	-1.1455613	0.6102134
11	C	-0.3575395	-1.3764043	1.4523008
12	H	0.1353716	-2.3446343	1.3688409
13	C	0.1173429	-0.3885788	2.3445123
14	C	-0.5762091	0.8676159	2.4080835
15	C	-1.7093219	1.0670151	1.5729374
16	H	-2.9813087	-3.915482	-2.0022206
17	H	-5.4747291	0.3831521	-1.8838635
18	H	-2.2388643	2.0198492	1.6180848
19	C	-0.1208069	1.8611766	3.2876052
20	C	1.2398378	-0.5810102	3.1741099
21	H	1.772507	-1.5326617	3.1318022

22	C	1.6860221	0.4439128	4.0624112
23	H	2.5597418	0.2636027	4.6910863
24	C	1.0211873	1.6456907	4.120647
25	C	-5.1175453	-3.831209	-3.7241714
26	H	-4.5730963	-4.7772278	-3.754526
27	C	-6.5043764	-1.3979081	-3.636352
28	C	-6.2168081	-3.6291593	-4.5296318
29	H	-6.5511793	-4.417489	-5.2064338
30	C	-6.9146489	-2.4031874	-4.4849858
31	H	-7.7850176	-2.253615	-5.1266612
32	H	-7.0440883	-0.4491255	-3.6010415
33	H	-0.652029	2.8140864	3.3314468
34	H	1.3525335	2.435661	4.7960395
35	O	-1.3417269	-3.2883783	-0.3292803
36	C	-5.9498128	-0.0503755	2.6354005
37	C	-4.8725536	-0.2286178	1.7783923
38	C	-4.5607913	0.6993108	0.7819599
39	C	-5.352121	1.8513115	0.6483398
40	C	-6.4478795	2.028116	1.5131236
41	C	-6.7400251	1.0885343	2.4909524
42	C	-3.9029961	2.7332358	-1.1024465
43	C	-3.0612654	1.6118765	-1.0294591
44	C	-1.902714	1.5787608	-1.8090081
45	H	-1.2588525	0.6993609	-1.7421107
46	C	-1.5636151	2.6189137	-2.6632905
47	C	-2.4086341	3.7244385	-2.7418668
48	C	-3.5621033	3.7842868	-1.9735635
49	H	-6.1722948	-0.7922765	3.4030479
50	H	-4.248558	-1.1201619	1.8688241
51	H	-7.0720461	2.9145087	1.4133177
52	H	-7.5962218	1.2531189	3.1477331
53	H	-0.6542542	2.5657963	-3.2628221
54	H	-2.1698598	4.5553272	-3.4082977
55	H	-4.2117656	4.6551419	-2.0430037
56	C	-5.9086729	3.9473397	-0.4535305
57	C	-5.6705893	5.0860994	0.317661
58	C	-6.9768105	3.9224308	-1.3497624
59	C	-6.5008393	6.1966757	0.191231
60	H	-4.8305481	5.0888579	1.0139895
61	C	-7.8060489	5.034737	-1.4746787
62	H	-7.1477158	3.0238618	-1.9444898
63	C	-7.5689547	6.1720619	-0.7047648
64	H	-6.3130595	7.0862365	0.7951042

65	H	-8.6414815	5.0126812	-2.1766066
66	H	-8.2192856	7.0430781	-0.8028277
67	N	-5.0631989	2.8077636	-0.3279834

Optimized STC ($^{1}LE_{A\_n\pi*}$/$^{3}LE_{A\_\pi\pi*}$) geometry of compound **ACRSP**

Center Number	Atomic Number	Coordinates (Angstroms)		
		X	Y	Z
1	C	-4.6665088	-2.8379748	-2.8478709
2	C	-3.5172355	-3.0156972	-1.9885461
3	C	-3.0862625	-1.9649241	-1.100902
4	C	-3.7954749	-0.7351201	-1.064677
5	C	-4.8919888	-0.5745538	-1.888742
6	C	-5.3592056	-1.6122072	-2.7995872
7	C	-1.9509239	-2.1733745	-0.2679792
8	C	-3.389242	0.4311625	-0.1404979
9	C	-2.153097	0.1169921	0.7238213
10	C	-1.4729342	-1.1448425	0.6475905
11	C	-0.3595559	-1.3891944	1.4444985
12	H	0.1443498	-2.3418803	1.3789701
13	C	0.1312899	-0.3984452	2.3517982
14	C	-0.5309346	0.835432	2.4289352
15	C	-1.6778661	1.067321	1.597976
16	H	-2.9708243	-3.9446815	-2.0072213
17	H	-5.4393911	0.3558346	-1.8700943
18	H	-2.1744801	2.0245026	1.6718446
19	C	-0.0488909	1.8216322	3.3267377
20	C	1.2653421	-0.6301387	3.172048
21	H	1.7727166	-1.5842199	3.108519
22	C	1.7118606	0.3338398	4.0292424
23	H	2.5775996	0.150292	4.6519793
24	C	1.0445938	1.5778384	4.1072661
25	C	-5.1067763	-3.8379182	-3.7175849
26	H	-4.5758184	-4.7804316	-3.7560996
27	C	-6.4698222	-1.4214496	-3.6202254
28	C	-6.2119506	-3.632617	-4.5263893
29	H	-6.5423315	-4.4160782	-5.1958321
30	C	-6.8955923	-2.4224398	-4.4785023
31	H	-7.7593089	-2.2609894	-5.1100377
32	H	-7.0028729	-0.4798783	-3.58387
33	H	-0.5609709	2.7734501	3.3852659
34	H	1.4053307	2.3364307	4.7895736
35	O	-1.3331138	-3.2955333	-0.3248975
36	C	-5.9785543	-0.0293591	2.6251327

37	C	-4.9032578	-0.2022246	1.788716
38	C	-4.5704232	0.7320736	0.7912549
39	C	-5.3723162	1.8750507	0.6490354
40	C	-6.4694979	2.0419897	1.5066002
41	C	-6.7684479	1.1081593	2.4761744
42	C	-3.918841	2.7603394	-1.1076599
43	C	-3.0701396	1.645863	-1.0218317
44	C	-1.9143929	1.6183254	-1.8231309
45	H	-1.2601981	0.7594465	-1.7594319
46	C	-1.5906137	2.6433948	-2.6776431
47	C	-2.439233	3.7450816	-2.755927
48	C	-3.5808979	3.8013999	-1.9845821
49	H	-6.2061828	-0.7660505	3.3835375
50	H	-4.2873322	-1.0844531	1.8983592
51	H	-7.0971755	2.9126852	1.4112455
52	H	-7.6227218	1.2703562	3.1203875
53	H	-0.6930592	2.5920642	-3.2788641
54	H	-2.2100299	4.5672353	-3.4212301
55	H	-4.2212885	4.6643404	-2.0647136
56	C	-5.9423952	3.9724973	-0.4682981
57	C	-5.7168145	5.1134068	0.2935254
58	C	-7.0048824	3.9437983	-1.3618723
59	C	-6.5481958	6.2162336	0.1617601
60	H	-4.8878056	5.130794	0.9881528
61	C	-7.8373018	5.0474335	-1.494101
62	H	-7.1755433	3.0536974	-1.9518859
63	C	-7.6103534	6.1850854	-0.7326762
64	H	-6.3664113	7.1006963	0.7580993
65	H	-8.6631073	5.017051	-2.1926563
66	H	-8.2587085	7.0453483	-0.8352893
67	N	-5.0895116	2.8341728	-0.3363396

Optimized STC ($^3LE_{A\_\pi\pi*}$/$^1CT_{\pi\pi*}$) geometry of compound **ACRSP**

Center Number	Atomic Number	Coordinates (Angstroms)		
		X	Y	Z
1	C	-4.6701689	-2.8836775	-2.7947531
2	C	-3.5697085	-3.0589012	-1.8944631
3	C	-3.1241615	-2.0074316	-1.0532383
4	C	-3.783863	-0.7511424	-1.1105004
5	C	-4.8420731	-0.5800491	-1.9719014
6	C	-5.3184744	-1.6323359	-2.8398316
7	C	-2.0075856	-2.2334049	-0.1647654
8	C	-3.3798454	0.4270529	-0.1984445

9	C	-2.149621	0.1301026	0.6869379
10	C	-1.5200161	-1.1604016	0.6867609
11	C	-0.4251998	-1.3818381	1.5168223
12	H	0.0445702	-2.3536535	1.5087346
13	C	0.0819441	-0.3621405	2.3677005
14	C	-0.5389336	0.9010634	2.36814
15	C	-1.6616665	1.1134155	1.5080693
16	H	-3.0552428	-4.0059688	-1.8480456
17	H	-5.3453116	0.3751247	-2.0217803
18	H	-2.1276194	2.0898978	1.5166014
19	C	-0.0362822	1.9218178	3.2149358
20	C	1.1998202	-0.5761992	3.2213291
21	H	1.6780394	-1.5472454	3.2213697
22	C	1.663559	0.421635	4.0266908
23	H	2.5146757	0.2466693	4.6722327
24	C	1.0374134	1.6916606	4.0254588
25	C	-5.1238322	-3.9166488	-3.6357716
26	H	-4.6277355	-4.8781851	-3.6052567
27	C	-6.396132	-1.4451152	-3.7166123
28	C	-6.1852356	-3.7133299	-4.4899037
29	H	-6.5237559	-4.5175767	-5.1301178
30	C	-6.8275531	-2.4708175	-4.5316642
31	H	-7.6603926	-2.3142133	-5.2046321
32	H	-6.8922417	-0.4831465	-3.7504434
33	H	-0.5174438	2.8920587	3.2097737
34	H	1.4145304	2.4764422	4.6682014
35	O	-1.4491162	-3.3702608	-0.144849
36	C	-6.006181	-0.1357564	2.4930337
37	C	-4.9271632	-0.2903339	1.6675761
38	C	-4.5748761	0.6823348	0.7120905
39	C	-5.3718957	1.8521399	0.6123986
40	C	-6.4875588	1.9943102	1.4720802
41	C	-6.7947009	1.0244148	2.3876503
42	C	-3.9097665	2.7703082	-1.1091985
43	C	-3.0812301	1.6444586	-1.0741425
44	C	-1.9541821	1.616936	-1.9064946
45	H	-1.3156325	0.7449193	-1.8841982
46	C	-1.6419148	2.6637022	-2.7432851
47	C	-2.4689593	3.7840083	-2.7659078
48	C	-3.5866684	3.8374065	-1.9614737
49	H	-6.2510469	-0.8983701	3.21914
50	H	-4.3208327	-1.1814297	1.7429054
51	H	-7.1041057	2.874785	1.4081459

52	H	-7.651627	1.1577524	3.0342866
53	H	-0.7643959	2.6140306	-3.3733154
54	H	-2.2420036	4.6198519	-3.4141538
55	H	-4.2144243	4.7121428	-1.9960201
56	C	-5.9040388	3.9969887	-0.3903532
57	C	-5.6529603	5.09974	0.4155141
58	C	-6.9652758	4.01506	-1.2843795
59	C	-6.4664257	6.2203541	0.3266967
60	H	-4.822289	5.0762421	1.1078545
61	C	-7.7778356	5.1373785	-1.3715928
62	H	-7.1509402	3.1515831	-1.9084439
63	C	-7.5296353	6.2404162	-0.5665322
64	H	-6.2689363	7.0780928	0.9557348
65	H	-8.6046319	5.1484512	-2.069197
66	H	-8.1633184	7.1146779	-0.634816
67	N	-5.065884	2.8362353	-0.3040345

Optimized SSC ($^{1}LE_{A\_n\pi*}/^{1}CT_{\pi\pi*}$) geometry of compound **ACRSP**

Center Number	Atomic Number	Coordinates (Angstroms) X	Y	Z
1	C	-4.7767244	-2.8598997	-2.6401927
2	C	-3.7003904	-3.0683888	-1.7307906
3	C	-3.1301935	-1.9830613	-0.9938439
4	C	-3.7285817	-0.6874239	-1.1365799
5	C	-4.7676326	-0.4854633	-2.0086861
6	C	-5.3174489	-1.5631347	-2.8030114
7	C	-1.9536256	-2.19369	-0.2454525
8	C	-3.2836841	0.4786445	-0.241647
9	C	-2.0728673	0.1720861	0.6483467
10	C	-1.4698869	-1.1381157	0.6489604
11	C	-0.4112903	-1.3829286	1.4917033
12	H	0.0368587	-2.3646432	1.4859717
13	C	0.0940608	-0.3858473	2.3596998
14	C	-0.4993414	0.8939202	2.3619238
15	C	-1.5909286	1.1386704	1.4845631
16	H	-3.2746236	-4.0518836	-1.6217186
17	H	-5.2183684	0.4902859	-2.1193235
18	H	-2.0364117	2.1252945	1.4990168
19	C	0.0110873	1.8962143	3.2291288
20	C	1.1881564	-0.6325757	3.2371766
21	H	1.646846	-1.6130177	3.2334173
22	C	1.6590865	0.3471643	4.0579488
23	H	2.4995989	0.1503152	4.7114447

24	C	1.0569464	1.6312003	4.0616465
25	C	-5.3259189	-3.9147157	-3.398093
26	H	-4.9162584	-4.9101169	-3.2825818
27	C	-6.364782	-1.3570051	-3.7075118
28	C	-6.3493633	-3.6874642	-4.2898005
29	H	-6.7423838	-4.507846	-4.8773884
30	C	-6.8936759	-2.4065009	-4.431851
31	H	-7.699064	-2.2328767	-5.1337094
32	H	-6.7714022	-0.3597614	-3.8253891
33	H	-0.4466576	2.87809	3.222576
34	H	1.4382339	2.3997437	4.7217646
35	O	-1.3437983	-3.3051263	-0.2823341
36	C	-5.9665414	-0.235296	2.3661285
37	C	-4.8444103	-0.3310945	1.5822171
38	C	-4.5097938	0.6759457	0.6377572
39	C	-5.3458108	1.8152477	0.5304908
40	C	-6.5218146	1.8952821	1.3554253
41	C	-6.8153408	0.9067984	2.2294592
42	C	-3.8509919	2.8141794	-1.1124723
43	C	-3.0018642	1.7174644	-1.089937
44	C	-1.8653881	1.734702	-1.892365
45	H	-1.2093014	0.875166	-1.881797
46	C	-1.5646372	2.817249	-2.6991563
47	C	-2.4113035	3.9144704	-2.7007433
48	C	-3.5487689	3.9172595	-1.9136175
49	H	-6.2095007	-1.009217	3.0790138
50	H	-4.1963176	-1.1890483	1.6661564
51	H	-7.1704784	2.7494733	1.2700535
52	H	-7.7074227	0.9817889	2.8370089
53	H	-0.6767771	2.8039225	-3.3159647
54	H	-2.1916564	4.7766054	-3.3163337
55	H	-4.1985734	4.776083	-1.9319767
56	C	-5.9236191	3.9577452	-0.4221787
57	C	-5.7385939	5.0463822	0.4173138
58	C	-6.9405872	3.9584959	-1.364342
59	C	-6.5822219	6.1434772	0.3144113
60	H	-4.9375415	5.032477	1.1438942
61	C	-7.7826745	5.0579206	-1.4643812
62	H	-7.068829	3.1043013	-2.0149688
63	C	-7.603989	6.1500079	-0.6262645
64	H	-6.4419971	6.9918545	0.9707792
65	H	-8.57717	5.0599991	-2.1985267
66	H	-8.2611934	7.0058502	-0.7052737

67	N	-5.0463826	2.8204381	-0.3248356

Optimized S_0 geometry of compound **ACRSA**

Center	Atomic	Coordinates (Angstroms)		
Number	Number	X	Y	Z
1	C	0.0000006	2.4860915	3.8902103
2	C	0.0000787	1.2742635	3.1752142
3	C	-0.00001	1.2843112	1.7734222
4	C	0.0000239	2.5288173	1.1171076
5	C	-0.0000563	0.0004367	3.9409483
6	C	-0.0000047	-0.0010687	0.9175418
7	C	-0.0000129	-1.2818525	1.7757662
8	C	0.0000697	-1.2694137	3.1696424
9	C	-0.0000719	-2.4910629	3.8862685
10	H	-0.000027	-2.451206	4.9655384
11	C	0.0000079	-2.527803	1.1181605
12	H	0.0000625	2.4453111	4.9693731
13	H	-0.0000094	2.5610156	0.0371794
14	H	-0.0000054	-2.5537627	0.0377278
15	O	0.0000416	-0.0028261	5.1498347
16	C	3.6965108	0.0006788	-0.0178749
17	C	2.5099979	-0.0004442	0.6829661
18	C	1.265649	-0.0009533	0.0480156
19	C	1.2213726	-0.0013986	-1.3465657
20	C	2.4324203	-0.0008968	-2.0615522
21	C	3.6452404	-0.0003341	-1.4071235
22	C	-1.2213703	-0.0014375	-1.3465757
23	C	-1.2656503	-0.000928	0.0480064
24	C	-2.5100118	-0.0003897	0.6829376
25	H	-2.5389415	-0.0012614	1.7638989
26	C	-3.6965176	0.0005046	-0.017911
27	C	-3.6452387	-0.0001896	-1.4071602
28	C	-2.4324109	-0.0008922	-2.0615779
29	H	4.6431323	0.0009739	0.504669
30	H	2.5389158	-0.0012275	1.7639269
31	H	2.4197506	-0.0015777	-3.1387764
32	H	4.5580523	0.0001551	-1.9888123
33	H	-4.643141	0.0009905	0.5046288
34	H	-4.5580463	-0.0000348	-1.9888561
35	H	-2.4197323	-0.0015607	-3.1388026
36	C	0.0000104	-0.0021524	-3.4627391
37	C	-0.0000589	-1.203271	-4.1608607
38	C	-0.0002456	1.1985071	-4.1617036

39	C	0.0000889	-1.2038906	-5.5488932
40	H	-0.0001144	-2.134774	-3.6111043
41	C	0.0004292	1.1982102	-5.5497084
42	H	-0.0000393	2.1303803	-3.6125833
43	C	-0.0002823	-0.0030843	-6.2455838
44	H	0.0004739	-2.1434315	-6.0857763
45	H	-0.0004427	2.1373908	-6.0872238
46	H	0.0000399	-0.0034546	-7.3277279
47	N	0.0000046	-0.0015972	-2.0339518
48	C	0.000167	3.6924818	3.229128
49	H	0.0000941	4.6182272	3.7897087
50	C	-0.0000129	3.7175514	1.8357113
51	H	0.0000228	4.662498	1.3084611
52	C	-0.0001678	-3.7024711	1.8199906
53	H	-0.0000612	-4.6463332	1.2903383
54	C	0.0000983	-3.6860188	3.2263181
55	H	-0.0000726	-4.6157964	3.7800052

Optimized $^3LE_{A\_n\pi*}$ geometry of compound **ACRSA**

Center Number	Atomic Number	Coordinates (Angstroms) X	Y	Z
1	C	-0.0002148	2.4863509	3.9098809
2	C	0.0000994	1.2636153	3.1851492
3	C	0.0000104	1.2906539	1.7720646
4	C	0.0000978	2.539525	1.1310613
5	C	0.0000418	0.003234	3.8537508
6	C	0.0000164	0.0045854	0.9130138
7	C	0.0000418	-1.2801516	1.7684164
8	C	0.0000488	-1.2578172	3.1719935
9	C	0.0000539	-2.491615	3.893529
10	H	0.0000024	-2.4744607	4.9744758
11	C	0.0000166	-2.5341955	1.1189305
12	H	0.0000889	2.4613291	4.9907855
13	H	0.0000061	2.5796513	0.0510605
14	H	0.0000113	-2.5640233	0.0384853
15	O	0.0000074	-0.0070554	5.2083476
16	C	3.6954837	0.004348	-0.0228738
17	C	2.5078673	0.0058411	0.6778023
18	C	1.2648242	0.0033953	0.0418237
19	C	1.2219826	-0.0015919	-1.3527952
20	C	2.4329739	-0.0015015	-2.0670083
21	C	3.6458486	0.0008572	-1.4114552
22	C	-1.2219621	-0.0015577	-1.3527913

23	C	-1.264793	0.0034168	0.0418284
24	C	-2.5078331	0.0058796	0.6778049
25	H	-2.5347986	0.0049073	1.7589345
26	C	-3.695453	0.0042304	-0.0228667
27	C	-3.6458239	0.0010177	-1.4114446
28	C	-2.432951	-0.0014846	-2.0670053
29	H	4.6416584	0.0043016	0.5003787
30	H	2.5348335	0.0049321	1.7589322
31	H	2.4219397	-0.004472	-3.1441522
32	H	4.5590449	-0.0009348	-1.9922822
33	H	-4.6416247	0.0044687	0.5003914
34	H	-4.5590231	-0.0010698	-1.992267
35	H	-2.4219251	-0.0044117	-3.1441503
36	C	0.0000226	-0.005493	-3.4691834
37	C	-0.0001233	-1.2080865	-4.1647455
38	C	-0.0001387	1.1936123	-4.170827
39	C	0.0001356	-1.2117716	-5.5527477
40	H	-0.0000666	-2.1382448	-3.6130504
41	C	0.0002675	1.1902882	-5.5588159
42	H	-0.000024	2.1265154	-3.6237733
43	C	-0.0002097	-0.0125072	-6.2520641
44	H	-0.0000503	-2.1522794	-6.0876301
45	H	-0.0000958	2.1280692	-6.0984631
46	H	0.0000663	-0.0152617	-7.3340587
47	N	0.000011	-0.0012678	-2.0406809
48	C	0.0003333	3.6871279	3.2589186
49	H	-0.0003786	4.60819	3.827009
50	C	-0.0002829	3.7273964	1.8555315
51	H	0.0000683	4.6765844	1.337285
52	C	0.0000987	-3.7047768	1.821366
53	H	0.000055	-4.6509157	1.2968822
54	C	0.0000557	-3.6798753	3.2326856
55	H	-0.0003413	-4.6075021	3.7898735

10. Reference.

[27] G. Pöschl, E. Teller, *Zeitschrift für Physik* **1933**, *83*, 143-151.

[28] a) C. Adamo, V. Barone, *J. Chem. Phys.* **1999**, *110*, 6158-6170; b) S. Grimme, J. Antony, S. Ehrlich, H. Krieg, *J. Chem. Phys.* **2010**, *132*; c) S. Grimme, S. Ehrlich, L. Goerigk, *J. Comput. Chem.* **2011**, *32*, 1456-1465.

[29] a) F. Neese, F. Wennmohs, U. Becker, C. Riplinger, *J. Chem. Phys.* **2020**, *152*; b) F. Neese, *WIREs Comput. Mol. Sci.* **2012**, *2*, 73-78.

[30] J. Tomasi, B. Mennucci, R. Cammi, *Chem. Rev.* **2005**, *105*, 2999-3094.

[31] M. J. Frisch, G. W. Trucks, H. B. Schlegel, G. E. Scuseria, M. A. Robb, J. R. Cheeseman, G. Scalmani, V. Barone, G. A. Petersson, H. Nakatsuji, X. Li, M. Caricato, A. V. Marenich, J. Bloino, B. G. Janesko, R. Gomperts, B. Mennucci, H. P. Hratchian, J. V. Ortiz, A. F. Izmaylov, J. L. Sonnenberg, Williams, F. Ding, F. Lipparini, F. Egidi, J. Goings, B. Peng, A. Petrone, T. Henderson, D. Ranasinghe, V. G. Zakrzewski, J. Gao, N. Rega, G. Zheng, W. Liang, M. Hada, M. Ehara, K. Toyota, R. Fukuda, J. Hasegawa, M. Ishida, T. Nakajima, Y. Honda, O. Kitao, H. Nakai, T. Vreven, K. Throssell, J. A. Montgomery Jr., J. E. Peralta, F. Ogliaro, M. J. Bearpark, J. J. Heyd, E. N. Brothers, K. N. Kudin, V. N. Staroverov, T. A. Keith, R. Kobayashi, J. Normand, K. Raghavachari, A. P. Rendell, J. C. Burant, S. S. Iyengar, J. Tomasi, M. Cossi, J. M. Millam, M. Klene, C. Adamo, R. Cammi, J. W. Ochterski, R. L. Martin, K. Morokuma, O. Farkas, J. B. Foresman, D. J. Fox, Wallingford, CT, **2016**.

[32] W. Humphrey, A. Dalke, K. Schulten, *J. Mol. Graph.* **1996**, *14*, 33-38.

[33] S. Battaglia, R. Lindh, *J. Chem. Theory Comput.* **2020**, *16*, 1555-1567.

[34] F. Weigend, *J. Comput. Chem.* **2008**, *29*, 167-175.

[35] V. Barone, M. Cossi, *J. Phys. Chem. A* **1998**, *102*, 1995-2001.

[36] a) F. Aquilante, J. Autschbach, A. Baiardi, S. Battaglia, V. A. Borin, L. F. Chibotaru, I. Conti, L. De Vico, M. Delcey, I. Fdez. Galván, N. Ferré, L. Freitag, M. Garavelli, X. Gong, S. Knecht, E. D. Larsson, R. Lindh, M. Lundberg, P. Å. Malmqvist, A. Nenov, J. Norell, M. Odelius, M. Olivucci, T. B. Pedersen, L. Pedraza-González, Q. M. Phung, K. Pierloot, M. Reiher, I. Schapiro, J. Segarra-Martí, F. Segatta, L. Seijo, S. Sen, D.-C. Sergentu, C. J. Stein, L. Ungur, M. Vacher, A. Valentini, V. Veryazov, *J. Chem. Phys.* **2020**, *152*; b) I. Fdez. Galván, M. Vacher, A. Alavi, C. Angeli, F. Aquilante, J. Autschbach, J. J. Bao, S. I. Bokarev, N. A. Bogdanov, R. K. Carlson, L. F. Chibotaru, J. Creutzberg, N. Dattani, M. G. Delcey, S. S. Dong, A. Dreuw, L. Freitag, L. M. Frutos, L. Gagliardi, F. Gendron, A. Giussani, L. González, G. Grell, M. Guo, C. E. Hoyer, M. Johansson, S. Keller, S. Knecht, G. Kovačević, E. Källman, G. Li Manni, M. Lundberg, Y. Ma, S. Mai, J. P. Malhado, P. Å. Malmqvist, P. Marquetand, S. A. Mewes, J. Norell, M. Olivucci, M. Oppel, Q. M. Phung, K. Pierloot, F. Plasser, M. Reiher, A. M. Sand, I. Schapiro, P. Sharma, C. J. Stein, L. K. Sørensen, D. G. Truhlar, M. Ugandi, L. Ungur, A. Valentini, S. Vancoillie, V. Veryazov, O. Weser, T. A. Wesołowski, P.-O. Widmark, S. Wouters, A. Zech, J. P. Zobel, R. Lindh, *J. Chem. Theory Comput.* **2019**, *15*, 5925-5964; c) G. Li Manni, I. Fdez. Galván, A. Alavi, F. Aleotti, F. Aquilante, J. Autschbach, D. Avagliano, A. Baiardi, J. J. Bao, S. Battaglia, L. Birnoschi, A. Blanco-González, S. I. Bokarev, R. Broer, R. Cacciari, P. B. Calio, R. K. Carlson, R. Carvalho Couto, L. Cerdán, L. F. Chibotaru, N. F. Chilton, J. R. Church, I. Conti, S. Coriani, J. Cuéllar-Zuquin, R. E. Daoud, N. Dattani, P. Decleva, C. de Graaf, M. G. Delcey, L. De Vico, W. Dobrautz, S. S. Dong, R. Feng, N. Ferré, M. Filatov, L. Gagliardi, M. Garavelli, L. González, Y. Guan, M. Guo, M. R. Hennefarth, M. R. Hermes, C. E. Hoyer, M. Huix-Rotllant, V. K. Jaiswal, A. Kaiser, D. S. Kaliakin, M. Khamesian, D. S. King, V. Kochetov, M. Krośnicki, A. A. Kumaar, E. D. Larsson, S. Lehtola, M.-B. Lepetit, H. Lischka, P. López Ríos, M. Lundberg, D. Ma, S. Mai, P. Marquetand, I. C. D. Merritt, F. Montorsi, M. Mörchen, A. Nenov, V. H. A. Nguyen, Y. Nishimoto, M. S. Oakley, M. Olivucci, M. Oppel, D. Padula, R. Pandharkar, Q. M. Phung, F. Plasser, G. Raggi, E. Rebolini, M. Reiher, I. Rivalta, D. Roca-Sanjuán, T. Romig, A. A. Safari, A. Sánchez-Mansilla, A. M. Sand, I. Schapiro, T. R. Scott, J. Segarra-Martí, F. Segatta, D.-C. Sergentu, P. Sharma, R. Shepard, Y. Shu, J. K. Staab, T. P. Straatsma, L. K. Sørensen, B. N. C. Tenorio, D. G. Truhlar, L. Ungur, M. Vacher, V. Veryazov, et al., *J. Chem. Theory Comput.* **2023**, *19*, 6933-6991.

[37] J. X. Zou, Molecular Orbital Kit (MOKIT), https://gitlab.com/jxzou/mokit (accessed Apr 13, 2024).

[38] E. Fermi, *Nuclear Physics*, University of Chicago Press, **1950**.

[39] a) S. H. Lin, *J. Chem. Phys.* **1966**, *44*, 3759-3767; b) S. H. Lin, W. Z. Xiao, W. Dietz, *Phys. Rev. E* **1993**, *47*, 3698-3706.

[40] S. Shaik, D. Danovich, R. N. Zare, *J. Am. Chem. Soc.* **2023**, *145*, 20132-20140.

[41] E. Espinosa, E. Molins, C. Lecomte, *Chem. Phys. Lett.* **1998**, *285*, 170-173.
[42] S. Emamian, T. Lu, H. Kruse, H. Emamian, *J. Comput. Chem.* **2019**, *40*, 2868-2881.
[43] K. Susumu, T. V. Duncan, M. J. Therien, *J. Am. Chem. Soc.* **2005**, *127*, 5186-5195.
